# Identification of small RNAs in extracellular vesicles from the commensal yeast *Malassezia sympodialis*

**DOI:** 10.1038/srep39742

**Published:** 2017-01-04

**Authors:** Simon Rayner, Sören Bruhn, Helen Vallhov, Anna Andersson, R. Blake Billmyre, Annika Scheynius

**Affiliations:** 1 Department of Medical Genetics, Oslo University Hospital and University of Oslo, Norway; 2 Translational Immunology Unit, Department of Medicine Solna, Karolinska Institutet and University Hospital Stockholm, Sweden; 3 Department of Clinical Science and Education, Karolinska Institutet, and Sachs’ Children and Youth Hospital, Södersjukhuset, SE-118 83 Stockholm, Sweden; 4 Department of Molecular Genetics and Microbiology, Duke University Medical Center, Durham, North Carolina, USA

## Abstract

*Malassezia* is the dominant fungus in the human skin mycobiome and is associated with common skin disorders including atopic eczema (AE)/dermatitis. Recently, it was found that *Malassezia sympodialis* secretes nanosized exosome-like vesicles, designated MalaEx, that carry allergens and can induce inflammatory cytokine responses. Extracellular vesicles from different cell-types including fungi have been found to deliver functional RNAs to recipient cells. In this study we assessed the presence of small RNAs in MalaEx and addressed if the levels of these RNAs differ when *M. sympodialis* is cultured at normal human skin pH versus the elevated pH present on the skin of patients with AE. The total number and the protein concentration of the released MalaEx harvested after 48 h culture did not differ significantly between the two pH conditions nor did the size of the vesicles. From small RNA sequence data, we identified a set of reads with well-defined start and stop positions, in a length range of 16 to 22 nucleotides consistently present in the MalaEx. The levels of small RNAs were not significantly differentially expressed between the two different pH conditions indicating that they are not influenced by the elevated pH level observed on the AE skin.

Extracellular vesicles (EV) are released not only from different mammalian cell-types but also from microorganisms and parasites and have the capacity to transfer complex biological information^1^,^2^,^3^,^4^,^5^. Various types of EV ranging in size from 20 nm to 1,000 nm in diameter have been described and are classified mainly on their mechanisms of biogenesis and their physiological functions^1^,^6^. Those designated exosomes are nanosized vesicles of 50–100 nm which are released extracellularly after fusion of multicellular endosomes with the cell membrane, whereas microvesicles (MV) are larger vesicles (100–1,000 nm) generated through outward budding of the plasma membrane^1^,^5^. Gram-negative bacteria produce MV by outward budding of the outer membrane and these vesicles are therefore referred to as outer membrane vesicles (OMV) with a diameter in the range of 20–500 nm^6^. Exosomes can be detected in body fluids such as urine, bronchoalveolar lavage fluid (BAL), breast milk and serum^7^. The functions of exosomes include immunoregulatory mechanisms such as modulation of antigen presentation, immune activation, immune suppression, immune surveillance and intercellular communication^6^. EV from microorganisms with thick cell walls, such as Gram-positive bacteria and fungi, have been associated with cytotoxicity, the invasion of host cells, and the transfer of virulence factors^2^. As seen with exosomes^1^,^8^, fungal EV have been observed to deliver functional messenger (m)RNAs and micro (mi)RNA-like RNAs to recipient cells^9^,^10^.

miRNAs are small non-coding RNAs with a length between 20 and 22 nucleotides (nt)^11^. They are spliced from precursor sequences that form the stable hairpin necessary for transportation from the nucleus to the cytoplasm. After the miRNA has been cleaved from this precursor, it is loaded into the RNA-induced silencing complex (RISC) which can bind to the 3′ untranslated region of an mRNA with partial sequence complementarity, leading to inhibition and degradation of the mRNA and producing post-transcriptional modification of gene expression levels^12^. miRNAs have been identified in humans^13^, plants^14^ and viruses^15^ and small RNAs with miRNA-like properties (milRNAs) have also been detected in the plant pathogens *Magnaporthe oryzae*^16^, *Sclerotinia sclerotiorum*^17^, *Botrytis cinerea*^18^ and *Phytophthora sojae*^19^, and in the filamentous fungi *Neurospora crassa*^20^. These milRNAs can play internal roles or, alternatively, impact host machinery. *S. sclerotiorum*, a plant pathogenic fungi, is an example of the former where it has been proposed that two milRNAs are involved in vegetative development^17^. Conversely, *B. cinerea*, an aggressive fungal pathogen that is able to infect more than 200 plant species, uses small RNAs to interfere with the host RNA interference (RNAi) machinery and selectively silences host immunity genes to achieve infection^18^. Furthermore, it was recently demonstrated that *Pseudomonas aeruginosa* is able to reduce the host immune response by releasing EVs containing small RNA that inhibit the IL-8 secretion of airway epithelial cells^21^. Thus, vesicle-mediated delivery of various cargo to host cells seem to be an important mechanism of host-pathogen communication and may play a major part in microbial pathogenesis.

*Malassezia* is a commensal yeast that colonizes the human skin right after birth and predominates the human fungal skin microflora^22^. Fourteen species have so far been identified on the skin of all warm blooded animals tested^23^. One of the species most frequently isolated from human skin is *Malassezia sympodialis*, which is associated with several common skin disorders such as atopic eczema (AE)/dermatitis^24^. AE is a complex inflammatory skin disorder that affects 15 to 20% of young children and up to 3% of adults^25^. Around 50% of adult AE patients are reactive to *M. sympodialis* in terms of specific IgE-and T-cell reactivity and/or positive atopy patch test (APT) reactions, indicating a link between AE and *M. sympodialis*^26^. Ten *M. sympodialis* allergens have been sequenced so far^27^. We have previously shown that *M. sympodialis* cultured at pH 6.1, which reflects the elevated skin pH of AE-patients, secrets more allergens compared to cultured at pH 5.5, which represents the normal skin pH^28^, suggesting a host-microbe interaction. Recently, we reported that *M. sympodialis* secretes nanosized exosome-like vesicles^29^. These vesicles, designated MalaEx, also carry allergens and can induce inflammatory cytokine responses with a significantly higher IL-4 production in peripheral blood mononuclear cells (PBMC) from patients with AE compared to healthy controls^29^. Thus, like human dendritic or B cell-derived exosomes^30^,^31^, MalaEx can participate in an allergic immune response^29^.

To elucidate *M. sympodialis* host-microbe interactions we here aimed to assess whether small RNAs are present in MalaEx and, if so, address whether the levels of these RNAs differ in MalaEx isolated from *M. sympodialis* cultured at normal skin pH compared to the higher pH on the skin of AE patients.

## Results

### Characterization of *M. sympodialis* cultured at different pH and of the isolated MalaEx

**Figure 1:**
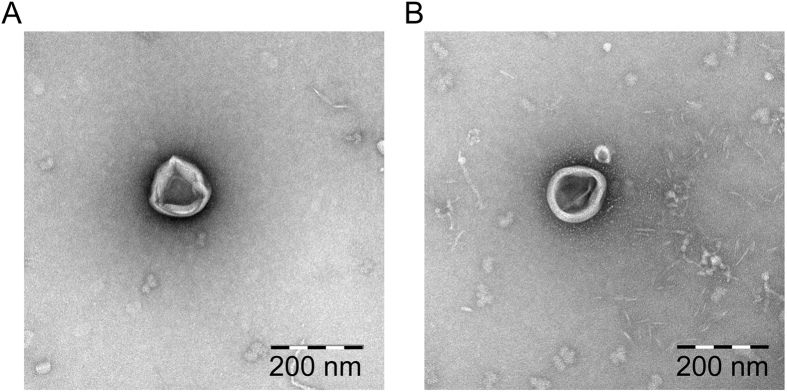
MalaEx visualized by TEM analyses. (**A**,**B**) MalaEx were isolated by ultracentrifugation followed by sucrose gradient centrifugation. Fractions ranging in density from 1.11–1.20 g/ml were pooled and analysed by TEM. Images show exosomes that derive from *M. sympodialis* which have been cultured at pH 5.5 (**A**) or at pH 6.1 (**B**). Scale bar indicates 200 nm.

The total number of *M. sympodialis* cells was similar between the two different pH conditions after 48 h culture (Table 1). The pH of the culture media slightly decreased between 0 h and 48 h with changes of 0.18 ± 0.01 for the cell-cultures at pH 5.5 and 0.14 ± 0.01 for the cell-cultures at pH 6.1. The size, the total number, and the protein concentration of the released MalaEx did not differ significantly between the two culture conditions (Table 1). The size range of the isolated MalaEx was 50–600 nm with a mean around 200 nm (Table 1). Transmission electron microscopy (TEM) analysis of sucrose gradient fractions revealed no significant morphological differences between MalaEx derived from cultures at pH 5.5 (Fig. 1A) compared with pH 6.1 (Fig. 1B).

### Identification of non-coding RNA features and differential expression analysis

From the 10 MalaEx samples isolated from 5 independent pairwise cultures at two different pH levels (Table 1) we first predicted non-coding features among the extracted RNA. This was done based on mapped reads that were present above different cutoffs, within different length intervals, and which had well defined start and stop positions. We found that the most stringent specifications (minimum counts 1000, 16 nt <= read length <= 25 nt) identified 325 non-coding features, of which three were predicted to be differentially expressed (DE) between the two pH conditions (Table 2). Conversely, the most relaxed conditions (minimum counts 50, 15 <= read length <= 30 nt) identified more than 2800 non-coding features of which 46 were predicted to be differentially expressed (Table 2). Using the annotated *M. sympodialis* genome that was sequenced and assembled with long-read technology^32^ sequencing (Zhu Y *et al*., manuscript submitted) we also investigated the fraction of reads that mapped to annotated (coding or ribosomal (r)RNA) and un-annotated (non-coding) regions. We found that ~55% of reads mapped to the non-coding regions (Supplementary Tables S1, S2 and S3). However, in all cases, the features that were predicted to be differentially expressed between the two pH conditions had extremely low read counts (<50), suggesting they have no biological significance and we therefore could not observe any significant differences in expression levels of identified non-coding features.

### Investigation of small RNA-like features in MalaEx

The annotated *M. sympodialis* genome (Zhu Y *et al*., manuscript submitted) only contains annotation for coding regions. We therefore investigated the mapped read set by considering whether any of the non-coding small RNA reads had any significant homology to identified small RNA classes in other similar genomes. To this end, we first considered *Ustilago maydis*^33^,^34^ as this is a well-studied basidiomycete fungus that is more closely related to *Malassezia* than other model ascomycete fungi such as *S. cerevisiae*. For comparative purposes, we also considered the reference genomes used by Peres *et al*.^9^ in their investigation of small RNA species in extracellular vesicles in fungi species. BLAST analysis using the identified feature set from MalaEx with reads >500 and lengths 15 to 30 nt as a query failed to identify any small RNA features that had significant overlap with any of the small RNA features from the five reference genomes. As an additional check, we also used BLAST to compare the sequence for the complete *M. sympodalis* genome (Zhu Y *et al*., manuscript submitted) against this reference database. We found significant hits to segments of several tRNA entries, as well as a single hit to a snRNA in *S. cerevisiae* (db_xref = SGD:S000006478), indicating that these features were present in the *M. sympodialis* genome. However, none of these features overlapped with the small RNA features we identified in the MalaEx NGS data.

### Characterization of the small RNA population in MalaEx

Although there were no highly expressed features that were predicted to be differentially expressed between the two pH conditions, many of the features were consistently expressed across almost all samples (i.e. at least 9 of the 10 pairwise cultured samples) and for both pH conditions (Supplementary Table S2). As a further check, we performed a correlation analysis of read count data for reads mapping to predicted small RNA features for all column wise comparisons. The results (Supplementary Fig. S1) show a strong correlation between almost all 10 samples, where even the lowest correlation values are within the range commonly seen in NGS data, supporting the argument that these reads are associated with expression of functional features, rather than random artifacts.

**Figure 2:**
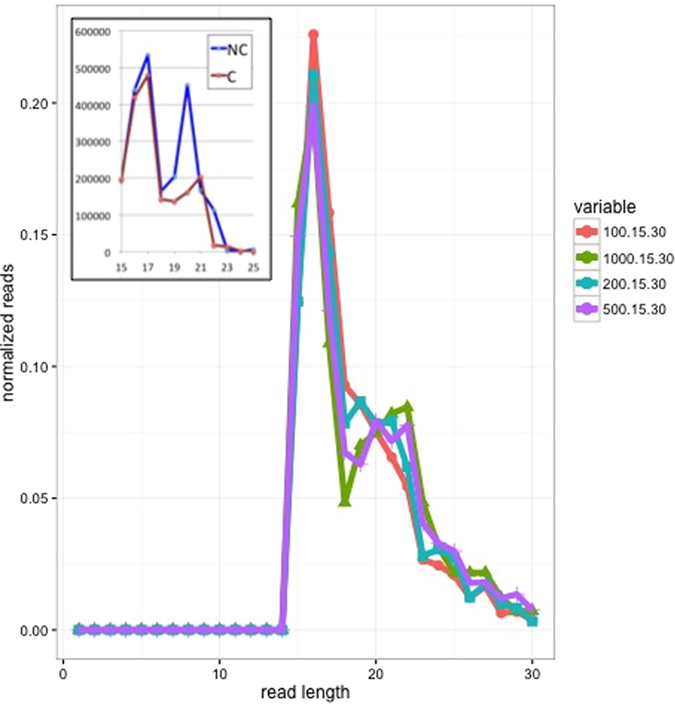
Length distribution of identified features for four different selection criteria based on read count and read length. (*i*) 100.15.30 (orange line: minimum read count for each feature is 100 nt, minimum read length is 15 nt, maximum read length is 30 nt), (*ii*) 1,000.15.30 (green line), (*iii*) 200.15.30 (blue line), and (*iv*) 500.15.30 (purple line). Each distribution shows a primary peak at 16 nt, and a secondary peak at 21 to 22 nt. The secondary peak is only visible with more stringent filtering (i.e. higher count cut off) and is not visible in the 100.15.30 dataset. Reads shorter than 15 nt were removed from the analysis. Insert. Reads map to coding or non-coding regions of the *M. sympodialis* genome according to the annotation from Zhu Y *et al*. (manuscript submitted). The mapped reads for the 500.15.30 annotation were summed over all samples and separated into coding (C, orange line) and non-coding (NC, blue line) groups and replotted. This graph shows that the secondary peak at 21 to 22 nt is strongly associated with the non-coding reads.

Based on this, we next investigated these features to determine whether we could establish if they were of functional significance, rather than a consequence of random events (such as degradation products). Figure 2 shows the feature length distribution for different filters according to read count and read length. For more stringent read count >= 500 reads, there is a distinct second peak present at a read length of 20 to 22 nt. Additionally, we separated the reads into those mapping to coding and non-coding regions and found that the second peak at 20–22 nt was strongly associated with the non-coding read set (Fig. 2 insert).

**Figure 3:**
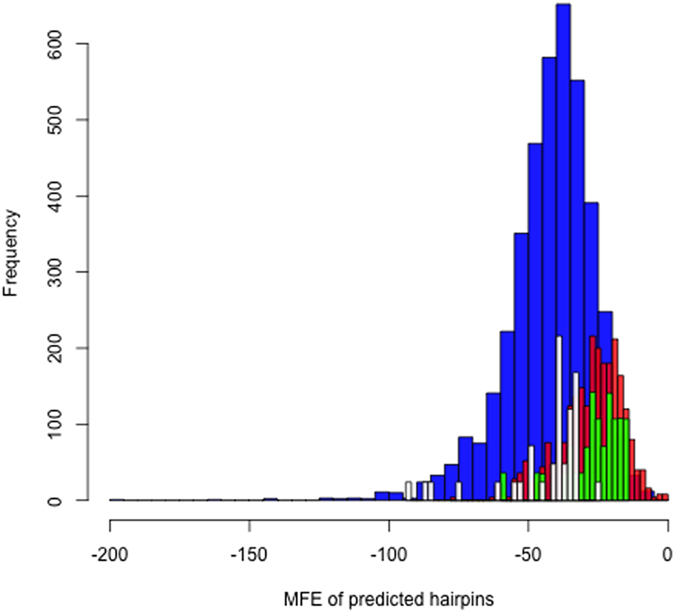
Mean free energy (MFE) distributions for (*i*) predicted hairpins from the identified MalaEx small RNA sequence set (*MalaEx:500:15:30*) with a 50 nt flanking region on each side (high count distribution at far right in red), (*ii*) predicted hairpins from randomly selected sequences from the *M. sympodialis* genome (Zhu Y *et al*., manuscript submitted; low count distribution at far right in green), (*iii*) human miRNA/pre-miRNAs from miRBase release 20 (largest distribution at far left, colored blue), and (*iv*) human cytomegalovirus (HCMV) miRNA/pre-miRNAs from miRBase release 20 shown in white. For readability, the frequency distributions for *M. sympodialis* and HCMV have been scaled by a factor of 4.

To investigate the possibility that the small RNAs were originating from an miRNA biogenesis-like pathway, we estimated the mean free energy (MFE) distribution of the complete small RNA set (*MalaEx:500:15:30*) based on the stability of predicted hairpin loops generated from each small RNA plus flanking sequence and compared this to the corresponding distributions for (*i*) randomly selected sequences from the *M. sympodialis* genome sequence (Zhu Y *et al*., manuscript submitted), (*ii*) human miRNAs, and (*iii*) human cytomegalovirus (HCMV) miRNAs (as an example of a non-canonical system) in miRBase release 20^35^. We found that the MFE distributions for *MalaEx* small RNAs and random sequence were most similar and notably less stable than the highly similar human and HCMV MFE distribution (Fig. 3). The profile of the MalaEx small RNA energy distribution is indistinguishable from the corresponding distribution for the randomly selected genome sequences. However, the median MFE energy is notably higher than the corresponding medians for known miRNAs/pre-miRNAs in human and HCMV (Fig. 3), indicating that the small RNA hairpins are unstable and unlikely to function as pre-miRNAs. Thus, given they do not form sufficiently stable precursor hairpins, it seems unlikely that MalaEx small RNA originate from a biogenesis miRNA biogenesis-like pathway.

We then investigated whether these small RNAs had complementary sequences in the human genome and found 56 features in the (*MalaEx:500:15:30*) set that mapped to the genome, but after filtering for a minimum average count >1000, only features *msy-10193* and *msy-4613* were above the threshold (Supplementary Table S4). However, TargetScan analysis^36^ of these two sequences failed to identify any targets, suggesting the overlap between these two genomes could be equally attributed to chance as to a functional role. Finally, we investigated the association between DNA methylation (m6A and m4C) and small RNA start position but were unable to show any enrichment of base modifications around the small RNA features in comparison to a set of randomly chosen loci (Supplementary Fig. S2).

## Discussion

In this study we aimed to investigate whether MalaEx are carriers of small RNAs and to address if the levels of these RNAs differ in MalaEx isolated from *M. sympodialis* cultured at normal skin pH versus the higher pH on the skin of AE patients. We did not find any significant differences between the MalaEx isolated between the two different pH levels regarding morphology. The size range of the isolated MalaEx was similar to other fungal EV such as those isolated from *Candida albicans*^37^. TEM analysis of MalaEx isolated using sucrose gradient fractions with density 1.11–1.20 g/ml used for exosomes^38^ revealed the presence of exosome-like vesicles as previously described^29^. The cellular origin of fungal EV and the mechanisms to transverse the thick cell wall remains unknown^10^,^39^. Future studies are needed to reveal the control of production and release of these vesicles. It has also to be remembered that the characterization of EV from different sources is still in need of technological advances for isolation and enrichment of the different subgroups of EV.

We then assessed the presence of small RNAs in MalaEx and addressed if the levels of these RNAs differ between the two pH conditions. Different mechanisms have been proposed for the generation of milRNAs in fungi. In *N. crassa*, milRNAs are generated from stem-looped precursors and require an RNase III domain containing protein (MRPL3), an exonuclease called QDE-2-interacting protein (QIP), an Argonaute-protein (QDE-2) and Dicer proteins^40^. The RNAi pathway is broadly conserved across eukaryotes, but surprisingly the 14 *Malassezia* species have recently been reported to lack homologs of the canonical RNAi pathway, including Dicer, Argonaute, and RNA-dependent RNA polymerase^41^. In spite of this, our analysis of the next generation sequencing data revealed a distribution of reads that was consistent with that observed in small RNA sequencing studies in miRNA expressing systems. Moreover, these patterns were revealed across all 10 MalaEx samples and in both pH conditions that we studied. Additionally, the predicted features were within a length range consistent with other small RNAs exhibiting a regulatory role and were mapped almost exclusively to non-coding regions. While two peaks were seen (Fig. 2) in the read length distribution (at ~16 nt and 21–22 nt) it is the second peak that is more interesting as this is consistent with miRNA populations, and is of the necessary length for binding to protein complexes in canonical pathways associated with small RNA function^42^.

A previous study of fungal extracellular vesicles examined both mRNAs and smaller size fractions (less than 200 bp) using Solid sequencing^9^. The authors identified 1,246 candidate miRNA sequences across four fungal species, not including *Malassezia*. Here we specifically examined RNAs smaller than 30 bp that were predicted to be highly enriched in mature miRNA or siRNA. While we also identified a candidate feature set, examination of the mean free energy suggested they were unlikely to form hairpin loops, providing evidence that miRNAs are not carried in *M. sympodialis* extracellular vesicles. It remains to be tested whether this is a biological difference or simply a result of differences in experimental and analytical approach.

One possible explanation for the biogenesis of these small RNAs is found in *N. crassa*. Dicer-independent small interfering RNAs (disiRNAs) originate from overlapping sense and antisense transcripts in *N. crassa*, but do not require any of the known RNA machinery^20^. These disiRNAs are capable of triggering DNA methylation that is enriched in the promotor regions of genes^43^. Production of small RNAs in *M. sympodialis* may represent a second example of disiRNA production, although our preliminary examination revealed no evidence for correlation of these small RNA features and base modifications (4mC and 6 mA, Supplementary Fig. S2). This could represent a biological difference between the function of RNAi-independent small RNAs in *M. sympodialis* and *N. crassa* or could be a limitation of our approach. An additional issue could simply be a matter of signal to noise; a large background of methylated bases could make the methylation linked to small RNAs very difficult to detect. Future experiments will be necessary to test whether presence of these small RNAs leads to increased DNA methylation in gene promoters as in *N. crassa*^43^. However, the continued presence of small RNAs with siRNA-like profiles in the absence of RNAi function is highly interesting in and of itself, particularly in the context of frequent, independent losses of RNAi across the eukaryotic tree of life (reviewed in refs 44 and 45). Further exploration of small RNA profiles in RNAi-deficient genomes may reveal a functional basis for the production of this class of small RNAs and may suggest an ancestral form of gene regulation based on overlapping sense and antisense RNAs that may have preceded the evolution of canonical RNAi in the common ancestor of the eukaryotic lineage.

There is precedent for small RNAs that are used by microorganisms to communicate across kingdoms with their hosts between fungi and plants^18^, intestinal nematodes and their mammalian hosts^46^, bacteria and humans^21^, and even in the reverse direction between sickle cell red blood cells and malaria parasites^47^. As a result, we explored whether the small RNAs found in MalaEx may also map to the human genome and represent cross-kingdom communication, possibly to modulate host immune response or to increase nutrient availability or to compete with other microbes on the ecological niche of human skin. Our TargetScan analysis^36^ failed, however, to identify any targets (Supplementary Table S4). An alternate possibility for these small RNAs is that they may play a role in either autocrine or paracrine signaling for *M. sympodialis*. In the latter case, one possibility is that quorum sensing could be mediated in a concentration dependent fashion by small RNA-containing MalaEx. Further experimental studies will be necessary to explore each of these hypotheses.

## Conclusions

This is the first characterization of small RNAs released from *M. sympodialis* via EV. We identified a set of reads with well-defined start and stop positions, in a length range of 16 to 22 nt, characteristic of small RNAs read distributions observed in other species that are loaded into EV. Bioinformatics analysis indicated that these RNA features appear to have an RNAi-independent route for biogenesis. No significant differences were observed between the MalaEx and their cargo of small RNAs isolated from *M. sympodialis* cultured at the two different pH levels. Thus, we did not find evidence that small RNA expression in MalaEx responds to a higher pH reflecting the level found on AE skin. The potential functional roles of these small RNAs carried by MalaEx remain to be elucidated.

## Methods

### *Malassezia sympodialis* culture

*M. sympodialis* (ATCC 42132) was cultured on Dixon agar plates^48^ modified to contain 1% (vol/vol) Tween 60, 1% (wt/vol) agar, and no oleic acid (mDixon) at 32 °C for 4 days before cells were harvested using a loophole. Cells were collected, dissolved in PBS and pelleted at 1.200× g for 5 min. The pellet was resuspended in PBS followed by sonication (5 × 20 sec) to obtain single cells. Cells were counted in a Burker chamber using trypan blue exclusion. 6 × 10^8^ cells were added to 300 ml mDixon broth supplemented with 50 mM MES (2-(N-Morpholino) ethanesulfonic acid) (Sigma Aldrich, St. Louis, Missouri, USA). The broth had been ultracentrifuged over night at 100.000× g and filtered through a 0.22 μm filter (Nordic Biolabs, Täby, Sweden) to remove possible nanovesicle contaminants. *M. sympodialis* was cultured pairwise at different pH values (pH 6.1 and 5.5) for 48 h at 32 °C and at 200 rpm. pH was measured at the start and end of culture using a pH-meter (Mettler Toledo, Greifensee, Switzerland). At each culture step blood and Sabourad agar plates were inoculated in parallel to exclude bacterial and *Candida* contaminations, respectively.

### MalaEx preparation

After 48 h culture *M. sympodialis* cells were separated by centrifugation at 1200× g for 5 min and the culture supernatant was used for the isolation of MalaEx by serial ultracentrifugation with an initial centrifugation of 3000 g for 30 min followed by a second at 10000× g for 30 min. Thereafter, MalaEx were pelleted at 100000 × g for 90 min, re-suspended in PBS and pelleted again at 100000× g for 90 min. The resulting pellet was carefully re-suspended in 100 μl PBS. Protein content was measured using a DC protein assay according to the manufacturer’s instructions (BioRad, Hercules, CA, USA). The MalaEx preparations were stored at −80 °C.

### NanoSight analysis

The particle size and concentration of the MalaEx preparations were measured using a LM10 platform with sCMOS camera from NanoSight Ltd, Amesbury, UK. The system is equipped with a 405 nm laser and was running the NTA 2.3 analytical software package. The samples were analyzed at 1000× dilution in PBS with camera level 14 and detection threshold 6. Two consecutive videos were recorded for each sample at room temperature.

### Transmission electron microscopy (TEM) - negative staining

Exosomes prepared for electron microscopy were further isolated by sucrose gradient centrifugation as previously described^49^. Fractions with a density of 1.10 to 1.20 g/ml were pooled from the MalaEx samples harvested from the cultures with the different pH values and aliquots of 3 μl were added to a grid with a glow discharged carbon coated supporting film for 3 minutes. The excess solution was soaked off by filter paper, the grid was rinsed in 5 μL distilled water for 10 seconds, stained with 2% uranyl acetate in water for 10 seconds and then air-dried. The samples were examined in a Hitachi 7700 electron microscope (Tokyo, Japan) at 80 kV and images were taken by a Veleta digital camera (Olympus Soft Imaging Solutions, GmbH, Münster, Germany).

### Extraction of RNA from MalaEx

RNA from MalaEx harvested from *M. sympodialis* cultured pairwise at pH 5.5 or pH 6.1 for 48 h was extracted from 5 different cultures with the miRCURY^TM^ RNA Isolation kit (Exiqon, Vedbaek, Denmark) using the specialized protocol for yeast cells according to the manufacturer’s instructions. RNA-concentration was measured with the Qubit Fluorometer 2.0 (Invitrogen, Carlsbad, CA, USA) and the RNA was stored at −80 °C until further usage.

### Small RNA-sequencing

RNA-sequencing was performed by Exiqon (Vedbæk, Denmark). As starting-material, one μg of RNA from 5 different independent pairwise cultures at pH 5.5 and pH 6.1 was utilized for the library generation. The assessment of the RNA quantity was done with a Bioanalyzer system (Agilent, Santa Clara, CA, USA). An automated gel cutter (LabChip XT, Perkin Elmer, Waltham, MA, USA) was used to excise the band that represents the microRNA-fraction. The cDNA libraries of small RNAs were constructed and then sequenced on a HiSeq 2500 (Illumina, San Diego, CA, USA).

### Data analysis

Reads were adapter trimmed using Trimmomatic version 0.34^50^ and trimmed reads were mapped to the abundant reference sequence set downloaded from the Illumina iGenome site (https://support.illumina.com/sequencing/sequencing_software/igenome.html). rRNA sequences within the *M. sympodalis* genome were obtained by BLASTing a PacBio assembled and annotated reference genome (Zhu Y *et al*., manuscript submitted) against the *Saccharomyces cerevisiae* reference genome (GCF_000146045.2_R64) downloaded from RefSeq and reads were further filtered against these sequences. Remaining reads were then mapped to the *M. sympodalis* genome (Zhu Y *et al*., manuscript submitted) using the bowtie alignment software package^51^.

To predict potential small RNA features, a modified version of miRPara^52^ was used to parse the generated SAM alignment files and examine the proximity of mapped reads to annotated coding regions. Reads that mapped to non-coding regions (i.e. outside regions with an “exon” annotation) in the *M. sympodialis* genome (Zhu Y *et al*., manuscript submitted) were then further examined to identify whether they exhibited “miRNA-like” profiles (i.e., well defined start and stop positions with the possibility of 5′ and 3′ modifications, and a length range and read distribution consistent with known miRNAs). Length intervals of 15 to 30 nt, 15 to 25 nt and 16 to 25 nt were examined, reads were further filtered by selecting a range of required minimum reads (50, 100, 200, 500 & 1,000) with the additional constraint that start and stop positions could each only vary by 4 nt. For each of these cases, a feature set was generated and used for counting reads intersecting each feature and then the EdgeR package^53^,^54^ was used to “normalize” consolidated counts and identify differentially expressed features.

### Investigation of small RNA features in the *M. sympodialis* genome

As our genome annotation of *M. sympodialis* (Zhu Y *et al*., manuscript submitted) doesn’t include annotation for snRNAs, snoRNAs or tRNAs, we performed the following analysis to investigate whether any of our identified features are mapping to sequences that have high similarity to any of these classes of small RNAs. We selected *U. maydis* (GCA_000328475.2) as the closest annotated genome to *M. sympodialis*^33^,^34^ as well as the four genomes studied by Peres *et al*.^9^, namely: (*C. neoformans* - GCA_000149245.3, *C. albicans* - GCA_000149445.2, *P. brasiliensis* - GCA_000150735.1 and *S. cerevisiae* - GCA_000146045.2).

We then extracted the sequences corresponding to identifiers with keywords equal to snRNA, snoRNA, tRNA and misc_RNA. This returned a total of 145 features corresponding to tRNA. Similarly, *C. neoformans* and *P. brasiliensis* returned 134 and 103 tRNA features, respectively. The remaining genomes had additional small RNA forms in their annotation: *C. albicans* (75 snoRNA, 5 snRNA, 5 other RNA); *S. cerevisiae* (77 snoRNA, 6 snRNA, 17 other RNA). For all these features, the corresponding sequence was extracted and used to build a BLAST reference small RNA database. The identified feature set from MS with reads >500 and lengths 15 to 30 nt were then BLASTed against this database using the *blastn_short* setting for short sequence matching.

### Characterization of identified small RNA features

To investigate whether a sub-population of these features might be achieving a regulatory role in their human host, we attempted to map the features to a human reference genome (release GRch37.p13, NCBI RefSeq accession number GCF_000001405.25). We selected the features set identified for a read cut off of 500 nt and a length range of 15 to 30 nt, and allowed for 2 mismatches (to accommodate for similar mismatches that are observed to be present in the seed region of miRNA targets). If these small RNAs were consistent with generation from a miRNA biogenesis-like pathway, we would expect to find they were part of a stable hairpin structure. We therefore selected a 50 bp-flanking region from both sides of each small RNA and predicted the secondary structure. If the predicted structure formed a hairpin, we recorded the MFE. After all small RNAs had been analysed, we plotted the MFE distribution for the set. We also performed a similar analysis for a set of sequences randomly selected from the genome sequence. Finally, we repeated the analysis for miRNA/pre-miRNAs in miRBase release 20 for *Homo sapiens* and for human cytomegalovirus (HCMV). We then compared the MFE distributions for the four datasets.

### Prediction of base modifications

Base modifications were predicted using PacBio data produced for *M. sympodialis* (Zhu Y *et al*. manuscript submitted). Data were aligned to the MS-PB reference genome using the SMRTAnalysis pipeline to uncover predicted modified bases as previously described^55^. The location of these predicted bases was compared to that of the small RNA features described above. As a control, three random sets of genomic loci were chosen of the same size as the small RNA data set, and base modifications were compared to those random loci as well.

### Data access

The data have been submitted to the Sequence Read Archive (SRA) database under study accession number BioProject ID PRJNA342612. https://www.ncbi.nlm.nih.gov/bioproject/342612.

## Additional Information

**How to cite this article**: Rayner, S. *et al*. Identification of small RNAs in extracellular vesicles from the commensal yeast *Malassezia sympodialis. Sci. Rep.* **7**, 39742; doi: 10.1038/srep39742 (2017).

**Publisher's note:** Springer Nature remains neutral with regard to jurisdictional claims in published maps and institutional affiliations.

## Supplementary Material

Supplementary Information

Supplementary Table S3

## Figures and Tables

**Table 1:** Characteristics of *M. sympodialis* cultures and isolated MalaEx after 48 h culture at different pH values.

Batch^a^	*M. sympodialis*			MalaEx		
	pH at start at harvest		Total cell number at harvest (×10^9^)	Mean vesicle size^b^ (nm)	Total no. of released vesicles^b^ (×10^12^)	Protein concentration^c^ (mg/ml)
A (n = 5)	5.5 ± 0.03	5.3 ± 0.01	201 ± 66	193.9 ± 9.9	249 ± 43.7	0.80 ± 0.26
B (n = 5)	6.1 ± 0.02	5.9 ± 0.04	261 ± 54	213.2 ± 12.0	164 ± 15	1.14 ± 0.52
p-value^d^			0.142	0.096	0.055	0.226

^a^All batches had a cell concentration of 2 × 10^6 ^cells/ml in 300 ml mDixon broth from start. ^b^The analysis was done using the LM 10-platform with sCMOS camera from NanoSight. ^c^The protein concentration was measured using a DC protein assay from BioRad. ^d^*P* values were calculated using a paired t-test. The values represent mean ± SD of five independent pairwise cultures.

**Table 2:** Number of predicted RNA features for different read cut off and length filters.

Min counts	Min length (nt)	Max length (nt)	No of features	No of DE features^a^
1000	15	30	414	3
1000	15	25	385	3
1000^b^	16	25	325	3
500	15	30	669	7
500	15	25	623	7
500	16	25	536	6
200	15	30	1211	12
200	15	25	1149	12
200	16	25	1016	12
100	15	30	1876	24
100	15	25	1790	23
100	16	25	1586	22
50^c^	15	30	2820	46
50	15	25	2699	44
50	16	25	2425	42

^a^Number of differential expression (DE) of small RNAs isolated from MalaEx harvested from 5 pairwise *M. sympodialis* cultures at pH 5.5 and pH 6.1, respectively (see Table 1). ^b^The most stringent specifications. ^c^Dataset selected for DE analysis and mapping to the human genome.
