# Supplementary material for: Identification of small RNAs in extracellular vesicles from the commensal yeast Malassezia sympodialis
Source: Sci Rep. 2017 Jan 4;7:39742. doi: 10.1038/srep39742 (PMC5209728; doi:10.1038/srep39742)
Supplement: Supplementary Information [file srep39742-s1.pdf]

Supplementary information

Identification of small RNAs in extracellular vesicles from the commensal yeast *Malassezia sympodialis*

Simon Rayner[1§], Sören Bruhn[2§], Helen Vallhov[3], Anna Andersson[2], R. Blake Billmyre[4] & Annika Scheynius[3*]

[1]Department of Medical Genetics, Oslo University Hospital and University of Oslo, Norway
[2]Translational Immunology Unit, Department of Medicine Solna, Karolinska Institutet and University Hospital Stockholm, Sweden
[3]Department of Clinical Science and Education, Karolinska Institutet, and Sachs' Children and Youth Hospital, Södersjukhuset, SE-118 83 Stockholm, Sweden
[4]Department of Molecular Genetics and Microbiology, Duke University Medical Center, Durham, North Carolina, USA

[§]These authors contributed equally to this work

[*]Corresponding author:

Annika Scheynius

Department of Clinical Science and Education
Södersjukhuset, Karolinska Institutet
SE-118 83 Stockholm, Sweden
Phone: +46 070 6057927

E-mail: annika.scheynius@ki.se

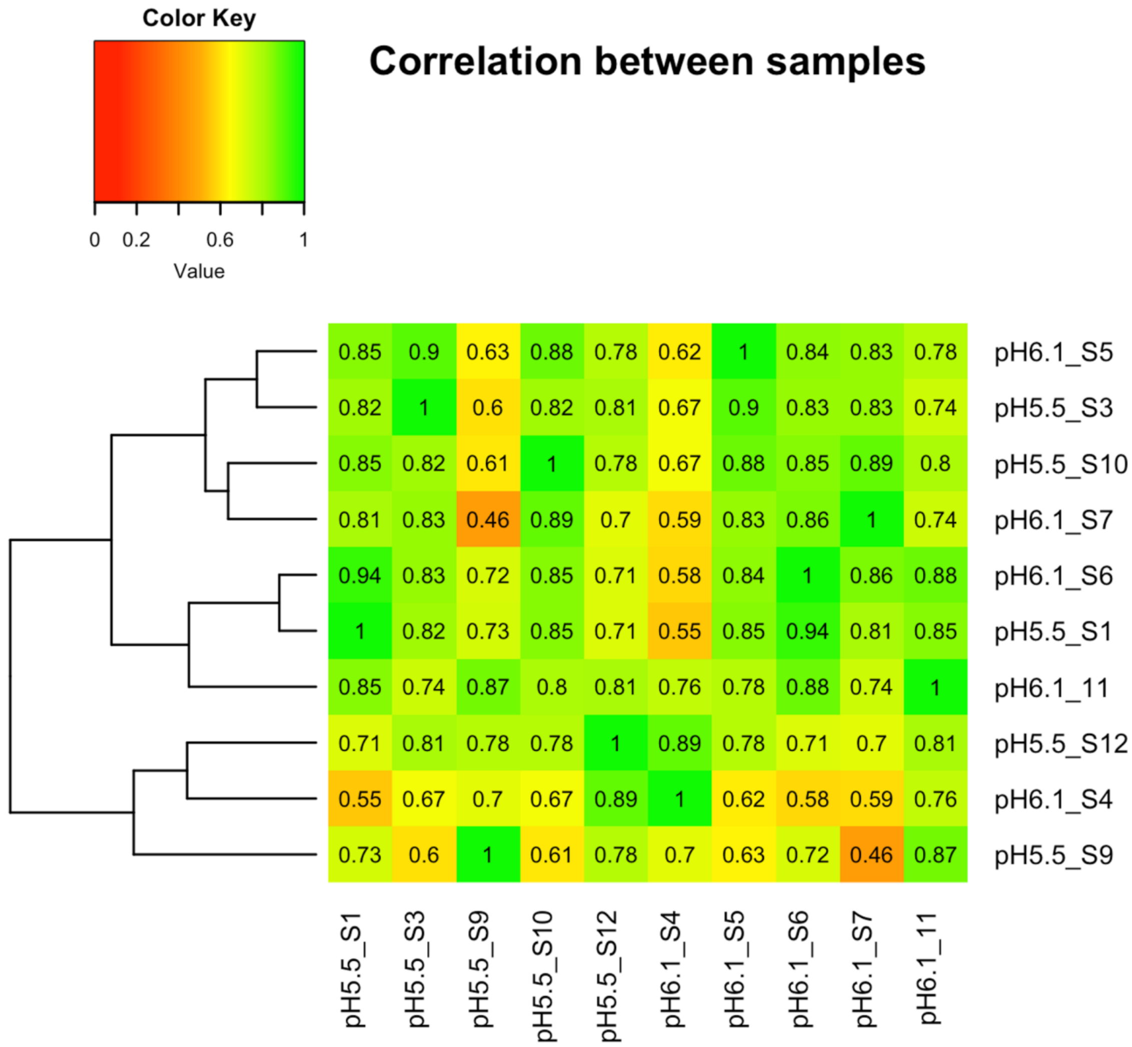

Supplementary Figure S1. Amongst correlation analysis of read count data for reads mapping to predicted small RNA features. Matrix was calculated for all column wise comparisons using the cor() function in R. The results show that there is a strong correlation between almost all samples, even the lowest correlation values are within the range commonly seen in NGS data, supporting our argument that these reads are associated with expression of functional features, rather than random artifacts.

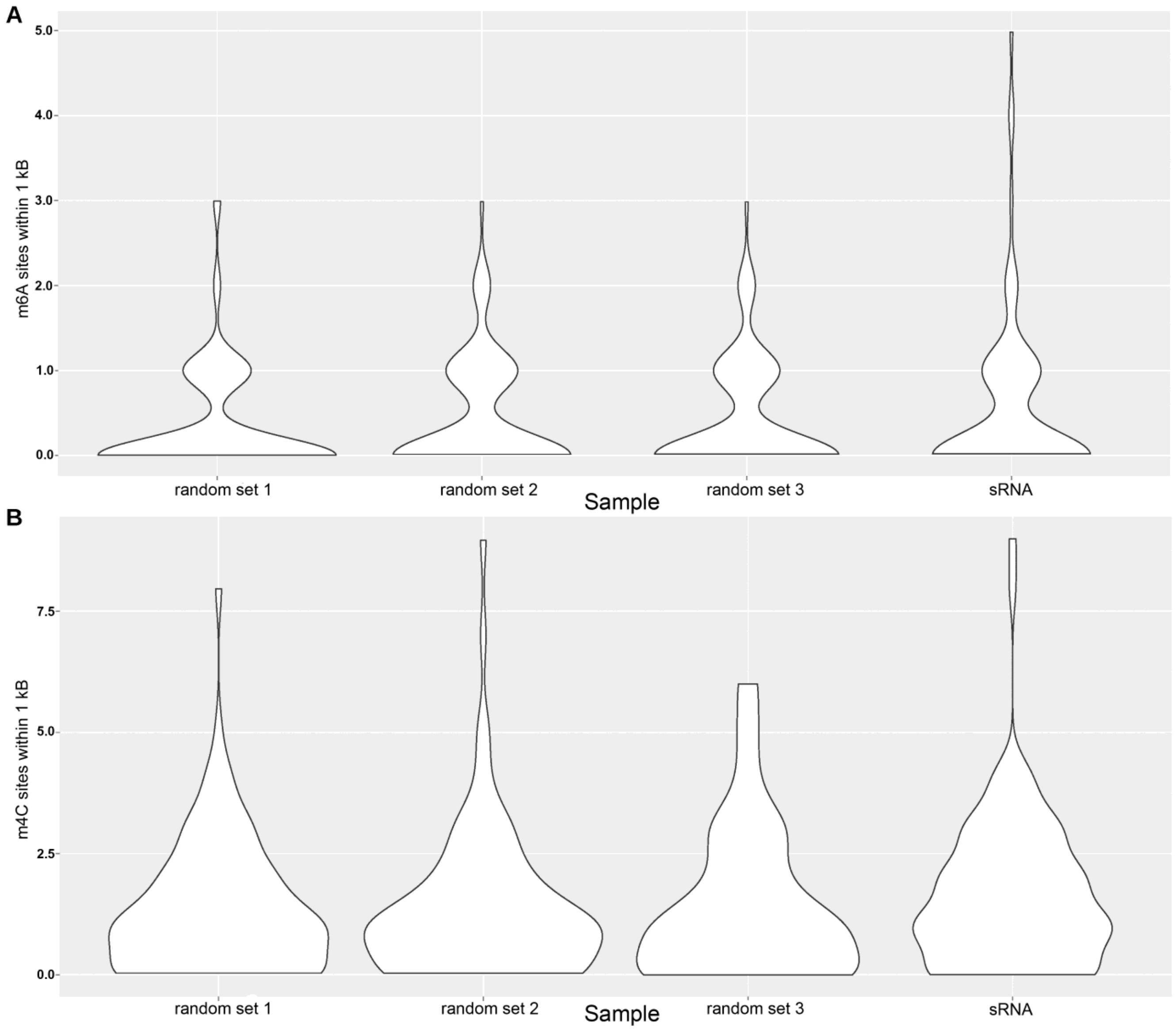

Supplementary Figure S2. Small RNA features are not enriched in predicted base modifications. The distributions of the counts of base modifications within a 1 kb window on either side of each predicted small RNA feature are plotted. Predicted m6A modifications (A) and m4C modifications (B) are each shown with three random sets of loci as controls.

Supplementary Table S1. Proportion of mapped reads that map to coding and non-coding regions of the MalaEx genome.

ID	Total reads	Mapped reads	Coding reads	Non-coding reads	% Non-coding reads
2726_S1.trim.clp.gen.count_summary.tsv	15611476	430732	192146	238586	55,4%
2726_S3.trim.clp.gen.count_summary.tsv	16468269	787209	351993	435216	55,3%
2726_S4.trim.clp.gen.count_summary.tsv	18118437	1153243	523355	629888	54,6%
2726_S5.trim.clp.gen.count_summary.tsv	22639058	1746786	781009	965777	55,3%
2726_S6.trim.clp.gen.count_summary.tsv	16206460	2183549	976495	1207054	55,3%
2726_S7.trim.clp.gen.count_summary.tsv	14592439	2532188	1131096	1401092	55,3%
2726_S9.trim.clp.gen.count_summary.tsv	17062248	2927582	1326635	1600947	54,7%
2726_S10.trim.clp.gen.count_summary.tsv	14561392	3384657	1511291	1873366	55,3%
2726_S11.trim.clp.gen.count_summary.tsv	15981161	3763591	1680899	2082692	55,3%
2726_S12.trim.clp.gen.count_summary.tsv	19983668	4168370	1873763	2294607	55,0%

Supplementary Table S2. Read counts for predicted features across all samples for a read cutoff of 500 (in at least one sample) and a length range from 16 to 25 nt (MalaEx:500:16:25), for pH 5.5 (columns 2 to 6) and pH 6.1 (columns 9 to 13). Average counts for the two conditions are shown in columns 7 and 14. These features map to coding or non-coding regions of the MalaEx genome according to the annotation from Zhu Y *et al* (manuscript submitted). The mapping is shown in column 15 (C: feature maps to coding region, NC: feature maps to non-coding region).

**SAMPLE name**	**pH 5.5 2726_S1**	**pH 5.5 2726_S3**	**pH 5.5 2726_S9**	**pH 5.5 2726_S10**	**pH 5.5 2726_S12**	**pH 5.5 AVERAGE**		**pH 6.1 2726_S4**	**pH 6.1 2726_S5**	**pH 6.1 2726_S6**	**pH 6.1 2726_S7**	**pH 6.1 2726_S11**	**pH 6.1 AVERAGE**	**LOCATION**
msy-206934:3|chr1_8977_8991|	55	89	21	162	106	86,6		225	137	82	253	125	164,4	NC
msy-48:5|chr1_40146_40165|	828	518	398	938	441	624,6		438	741	577	534	558	569,6	NC
Pmsy-110722:5|chr1_112430_112453[112429_112793]|	2	45	0	51	1	19,8		34	1	38	50	40	32,6	C
Pmsy-332362:5|chr1_126687_126708[126588_126712]|	0	0	0	0	0	0		0	1	0	0	0	0,2	C
Pmsy-250022:5|chr1_128456_128472[128107_129935]|	62	77	42	218	86	97		127	140	114	70	114	113	C
msy-570209:3|chr1_129129_129143|	81	41	79	88	51	68		41	90	68	40	60	59,8	NC
msy-63003:5|chr1_139147_139167|	122	89	53	93	94	90,2		79	111	107	181	74	110,4	NC
Pmsy-138811:3|chr1_150754_150769[149836_150881]|	327	173	170	320	134	224,8		121	312	297	163	148	208,2	C
Pmsy-83829:3|chr1_151621_151635[151562_152147]|	242	230	305	135	256	233,6		180	262	187	196	168	198,6	C

Pmsy-138853:3|chr1_159804_159818[158688_160367]|	68	30	47	86	159	78		136	245	84	47	164	135,2	C
msy-25336:5|chr1_164168_164190|	0	1	0	0	0	0,2		0	0	0	0	0	0	NC
Pmsy-239:5|chr1_164169_164188[163919_164614]|	755	527	319	645	473	543,8		433	842	765	834	484	671,6	C
msy-207817:3|chr1_169897_169911|	186	81	188	195	263	182,6		60	193	132	85	157	125,4	NC
msy-138954:3|chr1_182186_182201|	20	30	7	17	12	17,2		17	36	32	49	11	29	NC
msy-570661:3|chr1_182188_182203|	32	32	31	35	19	29,8		26	18	37	39	27	29,4	NC
Pmsy-111030:5|chr1_194437_194455[194387_194494]|	121	130	310	173	192	185,2		102	204	112	53	163	126,8	C
msy-41862:3|chr1_202342_202357|	283	183	49	180	135	166		17	345	231	128	146	173,4	NC
msy-300:5|chr1_208230_208249|	392	190	284	376	238	296		147	363	286	130	171	219,4	NC
Pmsy-63226:5|chr1_221847_221864[219760_221886]|	238	253	176	593	351	322,2		406	476	313	306	470	394,2	C
Pmsy-84190:3|chr1_260988_261004[260904_261007]|	387	386	228	490	312	360,6		329	602	356	443	528	451,6	C

<table>
<tr><th></th><th></th><th></th><th></th><th></th><th></th><th></th><th></th><th></th><th></th><th></th><th></th><th></th><th></th><th></th></tr>
<tr><td>Pmsy-286284:3|chr1_270538_270553[268631_271424]|</td><td>86</td><td>181</td><td>279</td><td>142</td><td>77</td><td>153</td><td></td><td>158</td><td>158</td><td>369</td><td>201</td><td>231</td><td>223,4</td><td>C</td></tr>
<tr><td>msy-208379:3|chr1_275228_275242|</td><td>81</td><td>80</td><td>41</td><td>109</td><td>99</td><td>82</td><td></td><td>113</td><td>180</td><td>99</td><td>125</td><td>101</td><td>123,6</td><td>NC</td></tr>
<tr><td>msy-111362:5|chr1_280485_280503|</td><td>202</td><td>151</td><td>86</td><td>232</td><td>98</td><td>153,8</td><td></td><td>102</td><td>154</td><td>143</td><td>164</td><td>97</td><td>132</td><td>NC</td></tr>
<tr><td>msy-208440:3|chr1_281969_281985|</td><td>153</td><td>146</td><td>77</td><td>219</td><td>112</td><td>141,4</td><td></td><td>154</td><td>144</td><td>161</td><td>201</td><td>137</td><td>159,4</td><td>NC</td></tr>
<tr><td>Pmsy-84276:3|chr1_281974_281989[281770_284725]|</td><td>5</td><td>10</td><td>7</td><td>13</td><td>9</td><td>8,8</td><td></td><td>10</td><td>22</td><td>12</td><td>14</td><td>10</td><td>13,6</td><td>C</td></tr>
<tr><td>Pmsy-571550:3|chr1_288037_288051[286940_290527]|</td><td>52</td><td>43</td><td>55</td><td>49</td><td>114</td><td>62,6</td><td></td><td>64</td><td>161</td><td>66</td><td>80</td><td>110</td><td>96,2</td><td>C</td></tr>
<tr><td>Pmsy-111406:5|chr1_295398_295414[294998_297880]|</td><td>206</td><td>116</td><td>125</td><td>118</td><td>0</td><td>113</td><td></td><td>90</td><td>177</td><td>176</td><td>152</td><td>0</td><td>119</td><td>C</td></tr>
<tr><td>msy-467080:3|chr1_308278_308293|</td><td>67</td><td>60</td><td>80</td><td>61</td><td>82</td><td>70</td><td></td><td>74</td><td>143</td><td>59</td><td>52</td><td>93</td><td>84,2</td><td>NC</td></tr>
<tr><td>msy-333287:5|chr1_318949_318968|</td><td>107</td><td>61</td><td>44</td><td>95</td><td>98</td><td>81</td><td></td><td>49</td><td>118</td><td>129</td><td>72</td><td>70</td><td>87,6</td><td>NC</td></tr>
<tr><td>Pmsy-208898:3|chr1_367926_367940[365610_369545]|</td><td>157</td><td>70</td><td>44</td><td>159</td><td>91</td><td>104,2</td><td></td><td>48</td><td>256</td><td>252</td><td>220</td><td>62</td><td>167,6</td><td>C</td></tr>
<tr><td>msy-84692:3|chr1_418108_418123|</td><td>3</td><td>368</td><td>414</td><td>633</td><td>462</td><td>376</td><td></td><td>464</td><td>1227</td><td>583</td><td>2</td><td>624</td><td>580</td><td>NC</td></tr>
</table>

msy-42394:3|chr1_423657_423673|	329	206	664	326	352	375,4		202	434	288	139	318	276,2	NC
msy-140181:3|chr1_447615_447629|	50	23	294	471	56	178,8		387	60	447	36	98	205,6	NC
msy-251375:5|chr1_449382_449403|	0	0	0	0	1	0,2		0	0	0	0	0	0	NC
Pmsy-176652:5|chr1_449382_449400[448724_449719]|	86	88	44	147	96	92,2		189	130	114	133	103	133,8	C
Pmsy-63764:5|chr1_453354_453376[451725_453986]|	0	1	0	2	0	0,6		0	0	1	2	0	0,6	C
msy-209415:3|chr1_475845_475859|	171	84	90	148	95	117,6		45	127	120	69	74	87	NC
Pmsy-112021:5|chr1_490549_490570[489490_491001]|	0	2	5	4	3	2,8		1	5	0	1	1	1,6	C
Pmsy-112062:5|chr1_501682_501700[501546_501784]|	208	98	188	76	171	148,2		60	250	146	135	96	137,4	C
msy-573155:3|chr1_502483_502498|	6	6	18	9	17	11,2		5	18	12	11	2	9,6	NC
msy-84959:3|chr1_502485_502499|	361	437	514	615	536	492,6		362	711	356	355	400	436,8	NC
Pmsy-11756:3|chr1_502552_502568[502007_503355]|	1818	3008	4654	629	3004	2622,6		3367	2666	1944	1836	1514	2265,4	C

Pmsy-176877:5|chr1_506912_506929[506735_507685]|	82	120	116	61	179	111,6		177	133	69	22	67	93,6	C
msy-112123:5|chr1_519603_519622|	305	154	160	208	121	189,6		102	193	309	234	105	188,6	NC
msy-525971:5|chr1_540123_540140|	0	0	0	0	0	0		0	0	0	0	1	0,2	NC
Pmsy-112186:5|chr1_540123_540138[539964_541316]|	150	139	110	207	180	157,2		156	366	163	153	255	218,6	C
msy-26053:5|chr1_553922_553940|	396	280	154	570	19	283,8		345	524	371	16	11	253,4	NC
msy-526069:5|chr1_559650_559666|	45	92	71	41	86	67		92	65	59	40	64	64	NC
msy-85195:3|chr1_567696_567710|	288	3	182	1	151	125		3	262	4	115	4	77,6	NC
msy-140724:3|chr1_567697_567712|	306	200	163	219	234	224,4		120	293	156	182	108	171,8	NC
msy-42928:3|chr1_626410_626425|	224	353	265	333	289	292,8		300	375	300	286	264	305	NC
Pmsy-334819:5|chr1_662991_663015[662972_663025]|	0	0	0	0	1	0,2		0	0	0	0	1	0,2	C
Pmsy-687803:3|chr1_665002_665016[664862_666028]|	72	37	30	40	54	46,6		29	88	64	44	98	64,6	C

Pmsy-425327:5|chr1_671827_671846[671724_671888]|	81	55	98	82	86	80,4		68	173	78	42	86	89,4	C
msy-526754:5|chr1_685068_685089|	0	0	0	0	1	0,2		0	0	0	0	1	0,2	NC
msy-858:5|chr1_685071_685088|	729	318	642	495	297	496,2		289	926	615	333	357	504	NC
Pmsy-112675:5|chr1_700054_700074[699390_702549]|	13	36	35	77	19	36		23	65	20	29	11	29,6	C
Pmsy-26333:5|chr1_707273_707291[707175_707327]|	397	321	3	3	482	241,2		2	743	1	303	849	379,6	C
Pmsy-637028:5|chr1_765025_765043[764060_765196]|	82	48	58	68	40	59,2		42	70	88	40	26	53,2	C
msy-113004:5|chr1_791186_791207|	0	1	1	0	1	0,6		1	2	1	2	0	1,2	NC
msy-85917:3|chr1_794397_794412|	353	114	220	251	121	211,8		74	300	288	154	146	192,4	NC
Pmsy-12217:3|chr1_794753_794768[794491_797688]|	940	743	669	1542	693	917,4		1069	1145	908	1264	807	1038,6	C
msy-141743:3|chr1_796361_796377|	270	144	162	184	125	177		74	217	217	158	106	154,4	NC
msy-64600:5|chr1_803543_803561|	339	3	231	421	3	199,4		801	511	329	7	6	330,8	NC

<table>
<tr><td></td><td></td><td></td><td></td><td></td><td></td><td></td><td></td><td></td><td></td><td></td><td></td><td></td><td></td><td></td></tr>
<tr><td>msy-113050:5|chr1_803543_803563|</td><td>0</td><td>0</td><td>0</td><td>0</td><td>0</td><td>0</td><td></td><td>0</td><td>1</td><td>0</td><td>0</td><td>0</td><td>0,2</td><td>NC</td></tr>
<tr><td>Pmsy-12248:3|chr1_806300_806315[806271_807938]|</td><td>1499</td><td>1623</td><td>2666</td><td>1441</td><td>1332</td><td>1712,2</td><td></td><td>997</td><td>1999</td><td>814</td><td>868</td><td>773</td><td>1090,2</td><td>C</td></tr>
<tr><td>msy-1023:5|chr1_807312_807330|</td><td>1018</td><td>821</td><td>491</td><td>1074</td><td>953</td><td>871,4</td><td></td><td>687</td><td>1002</td><td>1385</td><td>873</td><td>967</td><td>982,8</td><td>NC</td></tr>
<tr><td>msy-211234:3|chr1_818928_818942|</td><td>9</td><td>272</td><td>201</td><td>494</td><td>12</td><td>197,6</td><td></td><td>8</td><td>11</td><td>503</td><td>13</td><td>461</td><td>199,2</td><td>NC</td></tr>
<tr><td>Pmsy-64781:5|chr1_860857_860877[860837_863644]|</td><td>236</td><td>223</td><td>403</td><td>389</td><td>304</td><td>311</td><td></td><td>257</td><td>385</td><td>296</td><td>122</td><td>288</td><td>269,6</td><td>C</td></tr>
<tr><td>msy-113303:5|chr1_865988_866006|</td><td>346</td><td>2</td><td>204</td><td>6</td><td>7</td><td>113</td><td></td><td>3</td><td>292</td><td>375</td><td>212</td><td>2</td><td>176,8</td><td>NC</td></tr>
<tr><td>msy-689584:3|chr1_867365_867380|</td><td>94</td><td>48</td><td>54</td><td>55</td><td>43</td><td>58,8</td><td></td><td>33</td><td>64</td><td>63</td><td>87</td><td>38</td><td>57</td><td>NC</td></tr>
<tr><td>msy-26698:5|chr1_875232_875254|</td><td>201</td><td>121</td><td>71</td><td>206</td><td>107</td><td>141,2</td><td></td><td>76</td><td>207</td><td>244</td><td>197</td><td>125</td><td>169,8</td><td>NC</td></tr>
<tr><td>msy-64838:5|chr1_883626_883650|</td><td>60</td><td>57</td><td>96</td><td>40</td><td>72</td><td>65</td><td></td><td>47</td><td>79</td><td>79</td><td>35</td><td>68</td><td>61,6</td><td>NC</td></tr>
<tr><td>msy-471437:3|chr1_896267_896281|</td><td>13</td><td>105</td><td>16</td><td>20</td><td>10</td><td>32,8</td><td></td><td>94</td><td>17</td><td>10</td><td>5</td><td>112</td><td>47,6</td><td>NC</td></tr>
<tr><td>msy-12434:3|chr1_896269_896285|</td><td>10522</td><td>7663</td><td>23113</td><td>10790</td><td>11259</td><td>12669,4</td><td></td><td>7041</td><td>15270</td><td>10185</td><td>4223</td><td>9581</td><td>9260</td><td>NC</td></tr>
</table>

<table>
<tr><td>msy-142353:3|chr1_909709_909723|</td><td>165</td><td>83</td><td>122</td><td>119</td><td>108</td><td>119,4</td><td></td><td>80</td><td>224</td><td>102</td><td>93</td><td>100</td><td>119,8</td><td>NC</td></tr>
<tr><td>Pmsy-86420:3|chr1_918723_918738[917555_919264]|</td><td>233</td><td>213</td><td>140</td><td>248</td><td>409</td><td>248,6</td><td></td><td>347</td><td>483</td><td>315</td><td>409</td><td>457</td><td>402,2</td><td>C</td></tr>
<tr><td>msy-638155:5|chr1_926462_926483|</td><td>57</td><td>50</td><td>48</td><td>66</td><td>71</td><td>58,4</td><td></td><td>53</td><td>83</td><td>75</td><td>60</td><td>57</td><td>65,6</td><td>NC</td></tr>
<tr><td>msy-253590:5|chr1_951855_951878|</td><td>0</td><td>0</td><td>0</td><td>0</td><td>0</td><td>0</td><td></td><td>0</td><td>0</td><td>0</td><td>1</td><td>0</td><td>0,2</td><td>NC</td></tr>
<tr><td>Pmsy-113623:5|chr1_951856_951875[951481_952340]|</td><td>200</td><td>120</td><td>182</td><td>137</td><td>110</td><td>149,8</td><td></td><td>74</td><td>208</td><td>207</td><td>101</td><td>179</td><td>153,8</td><td>C</td></tr>
<tr><td>msy-142726:3|chr1_978491_978506|</td><td>189</td><td>132</td><td>223</td><td>208</td><td>153</td><td>181</td><td></td><td>124</td><td>328</td><td>228</td><td>140</td><td>153</td><td>194,6</td><td>NC</td></tr>
<tr><td>Pmsy-113828:5|chr1_1004835_1004852[1002899_1004859]|</td><td>159</td><td>135</td><td>175</td><td>122</td><td>137</td><td>145,6</td><td></td><td>128</td><td>287</td><td>91</td><td>59</td><td>110</td><td>135</td><td>C</td></tr>
<tr><td>Pmsy-86821:3|chr1_1038860_1038874[1038096_1042529]|</td><td>21</td><td>36</td><td>85</td><td>121</td><td>44</td><td>61,4</td><td></td><td>33</td><td>170</td><td>97</td><td>36</td><td>112</td><td>89,6</td><td>C</td></tr>
<tr><td>msy-113970:5|chr1_1045357_1045375|</td><td>159</td><td>137</td><td>84</td><td>174</td><td>150</td><td>140,8</td><td></td><td>147</td><td>159</td><td>179</td><td>231</td><td>108</td><td>164,8</td><td>NC</td></tr>
<tr><td>msy-12708:3|chr1_1063657_1063673|</td><td>3684</td><td>7641</td><td>126</td><td>36</td><td>130</td><td>2323,4</td><td></td><td>241</td><td>8767</td><td>4049</td><td>1661</td><td>69</td><td>2957,4</td><td>NC</td></tr>
<tr><td>Pmsy-291052:3|chr1_1065597_1065611[1064221_1065705]|</td><td>136</td><td>67</td><td>163</td><td>195</td><td>91</td><td>130,4</td><td></td><td>32</td><td>125</td><td>156</td><td>36</td><td>111</td><td>92</td><td>C</td></tr>
</table>

msy-691500:3|chr1_1076983_1076997|	1	0	1	2	0	0,8		0	0	1	1	5	1,4	NC
Pmsy-254217:5|chr1_1088140_1088168[1087971_1088436]|	0	0	1	3	4	1,6		0	7	3	2	5	3,4	C
msy-377403:3|chr1_1104500_1104514|	118	70	45	64	116	82,6		37	145	110	63	39	78,8	NC
Pmsy-143269:3|chr1_1107135_1107149[1106432_1108831]|	41	43	52	38	43	43,4		20	76	32	26	36	38	C
Pmsy-179417:5|chr1_1138688_1138710[1138625_1140053]|	39	31	22	33	27	30,4		21	41	41	22	23	29,6	C
Pmsy-12851:3|chr1_1157810_1157824[1157088_1158230]|	2757	185	1737	226	1769	1334,8		184	443	2440	267	2278	1122,4	C
Pmsy-44301:3|chr1_1167941_1167955[1167221_1168869]|	260	166	157	188	338	221,8		164	514	315	296	326	323	C
msy-578647:3|chr1_1184016_1184031|	1	200	107	3	239	110		2	1	163	1	167	66,8	NC
msy-639838:5|chr1_1191464_1191485|	1	1	1	0	0	0,6		0	0	1	1	1	0,6	NC
msy-473547:3|chr1_1196671_1196687|	37	88	53	66	67	62,2		138	80	51	58	64	78,2	NC
Pmsy-114472:5|chr1_1196917_1196936[1195298_1197758]|	421	50	766	68	391	339,2		35	502	380	163	372	290,4	C

msy-179673:5|chr1_1203243_1203258|	108	104	77	163	106	111,6		134	165	173	121	102	139	NC
Pmsy-12994:3|chr1_1249838_1249852[1249286_1250008]|	1519	961	834	1398	1196	1181,6		892	2112	1378	1091	991	1292,8	C
Pmsy-13006:3|chr1_1254893_1254908[1254585_1255322]|	1290	636	730	1424	551	926,2		653	1347	1142	605	700	889,4	C
msy-692915:3|chr1_1259025_1259041|	66	38	65	47	70	57,2		24	73	70	45	85	59,4	NC
Pmsy-27539:5|chr1_1278274_1278293[1275642_1278639]|	374	333	238	744	356	409		423	688	500	372	391	474,8	C
Pmsy-1730:5|chr1_1295081_1295100[1294011_1297355]|	1039	624	731	732	666	758,4		456	829	932	641	479	667,4	C
msy-579525:3|chr1_1297418_1297432|	26	66	30	40	6	33,6		19	54	42	34	21	34	NC
msy-1733:5|chr1_1297618_1297636|	640	1	489	388	1	303,8		239	3	9	1	393	129	NC
Pmsy-429155:5|chr1_1359273_1359296[1358725_1360200]|	0	0	0	2	0	0,4		0	0	0	0	1	0,2	C
msy-66095:5|chr1_1364888_1364905|	74	155	143	52	173	119,4		368	120	74	20	48	126	NC
msy-87916:3|chr1_1382294_1382309|	6	336	4	359	375	216		280	656	11	6	16	193,8	NC

Pmsy-13240:3|chr1_1384666_1384680[1384275_1385243]|	2806	908	1769	688	1049	1444		780	2246	1759	2449	1202	1687,2	C
msy-144495:3|chr1_1388877_1388891|	8	211	214	430	361	244,8		270	423	8	11	365	215,4	NC
Pmsy-27771:5|chr1_1397266_1397285[1396942_1400412]|	379	198	302	195	294	273,6		164	369	403	315	238	297,8	C
Pmsy-1867:5|chr1_1401875_1401900[1401336_1406087]|	0	0	0	1	0	0,2		1	0	1	0	0	0,4	C
Pmsy-13287:3|chr1_1407552_1407566[1406921_1408808]|	4522	4374	6812	2881	5443	4806,4		3190	5295	3111	2232	3047	3375	C
msy-580592:3|chr1_1428140_1428154|	141	77	111	3	0	66,4		1	108	5	82	62	51,6	NC
msy-180591:5|chr1_1428434_1428451|	0	111	1	0	0	22,4		84	172	316	225	148	189	NC
msy-379491:3|chr1_1436733_1436749|	130	103	242	124	147	149,2		85	218	132	65	120	124	NC
msy-13355:3|chr1_1450807_1450821|	424	320	383	422	326	375		270	855	407	198	427	431,4	NC
msy-13357:3|chr1_1450809_1450823|	1556	1049	1233	1561	793	1238,4		705	1890	1724	1030	875	1244,8	NC
Pmsy-115519:5|chr1_1488729_1488748[1488697_1490588]|	204	164	109	160	148	157		118	333	219	213	196	215,8	C

msy-13493:3|chr2_33318_33332|	471	20	280	25	15	162,2		22	29	519	443	373	277,2	NC
msy-88412:3|chr2_33320_33334|	868	612	481	746	691	679,6		556	1014	993	927	540	806	NC
msy-115686:5|chr2_35980_36000|	0	0	0	0	1	0,2		0	1	0	0	0	0,2	NC
Pmsy-2062:5|chr2_35981_35998[35955_36322]|	833	116	450	923	2	464,8		115	6	2	2	160	57	C
msy-215334:3|chr2_82463_82477|	192	38	26	62	86	80,8		88	49	151	123	122	106,6	NC
msy-13627:3|chr2_117502_117517|	783	473	599	1336	603	758,8		572	1187	807	428	784	755,6	NC
Pmsy-181565:5|chr2_157115_157132[155719_157620]|	113	106	126	147	72	112,8		82	217	171	104	120	138,8	C
Pmsy-696701:3|chr2_185458_185473[185315_188245]|	49	1	80	40	90	52		23	5	58	124	107	63,4	C
msy-181738:5|chr2_198623_198644|	1	1	5	3	2	2,4		5	3	0	1	2	2,2	NC
msy-28428:5|chr2_206777_206797|	216	494	268	413	356	349,4		506	436	299	277	264	356,4	NC
msy-13834:3|chr2_237328_237343|	4115	2244	2671	3106	1837	2794,6		1996	3856	3555	2380	2359	2829,2	NC
msy-116494:5|chr2_259533_259554|	125	92	56	176	105	110,8		116	203	102	129	126	135,2	NC

<table>
<tr><td>Pmsy-89195:3|chr2_273261_273277[263704_275229]|</td><td>181</td><td>277</td><td>154</td><td>241</td><td>350</td><td>240,6</td><td></td><td>335</td><td>293</td><td>284</td><td>484</td><td>227</td><td>324,6</td><td>C</td></tr>
<tr><td>Pmsy-45864:3|chr2_280956_280971[279572_281293]|</td><td>315</td><td>2181</td><td>923</td><td>2207</td><td>328</td><td>1190,8</td><td></td><td>1629</td><td>3748</td><td>22</td><td>1202</td><td>2061</td><td>1732,4</td><td>C</td></tr>
<tr><td>msy-340380:5|chr2_287434_287453|</td><td>148</td><td>135</td><td>87</td><td>243</td><td>8</td><td>124,2</td><td></td><td>14</td><td>10</td><td>11</td><td>246</td><td>248</td><td>105,8</td><td>NC</td></tr>
<tr><td>msy-340596:5|chr2_328768_328785|</td><td>98</td><td>59</td><td>52</td><td>102</td><td>73</td><td>76,8</td><td></td><td>50</td><td>113</td><td>123</td><td>116</td><td>84</td><td>97,2</td><td>NC</td></tr>
<tr><td>msy-2478:5|chr2_333541_333560|</td><td>669</td><td>629</td><td>799</td><td>586</td><td>436</td><td>623,8</td><td></td><td>470</td><td>1317</td><td>1001</td><td>529</td><td>600</td><td>783,4</td><td>NC</td></tr>
<tr><td>Pmsy-697942:3|chr2_333541_333556[332156_333562]|</td><td>4</td><td>4</td><td>8</td><td>3</td><td>124</td><td>28,6</td><td></td><td>55</td><td>167</td><td>6</td><td>106</td><td>96</td><td>86</td><td>C</td></tr>
<tr><td>Pmsy-28697:5|chr2_335774_335791[333616_337155]|</td><td>561</td><td>303</td><td>291</td><td>476</td><td>294</td><td>385</td><td></td><td>222</td><td>510</td><td>501</td><td>392</td><td>200</td><td>365</td><td>C</td></tr>
<tr><td>Pmsy-146596:3|chr2_358790_358804[357684_359489]|</td><td>234</td><td>90</td><td>205</td><td>182</td><td>157</td><td>173,6</td><td></td><td>175</td><td>167</td><td>317</td><td>128</td><td>382</td><td>233,8</td><td>C</td></tr>
<tr><td>Pmsy-14117:3|chr2_409392_409407[408945_410134]|</td><td>2069</td><td>84</td><td>1132</td><td>1890</td><td>1662</td><td>1367,4</td><td></td><td>112</td><td>2061</td><td>158</td><td>208</td><td>1602</td><td>828,2</td><td>C</td></tr>
<tr><td>msy-257938:5|chr2_411463_411482|</td><td>111</td><td>72</td><td>149</td><td>191</td><td>244</td><td>153,4</td><td></td><td>81</td><td>187</td><td>101</td><td>82</td><td>307</td><td>151,6</td><td>NC</td></tr>
<tr><td>Pmsy-2589:5|chr2_421196_421215[421034_422384]|</td><td>3291</td><td>3615</td><td>3000</td><td>1986</td><td>5009</td><td>3380,2</td><td></td><td>3961</td><td>4492</td><td>3108</td><td>3222</td><td>3077</td><td>3572</td><td>C</td></tr>
</table>

msy-479048:3|chr2_442336_442350|	3	75	83	92	1	50,8		37	134	145	1	1	63,6	NC
msy-382825:3|chr2_443187_443202|	100	131	131	111	35	101,6		52	131	84	65	53	77	NC
msy-46415:3|chr2_499485_499499|	407	256	319	298	325	321		205	466	325	255	337	317,6	NC
msy-14257:3|chr2_502523_502538|	541	1104	694	329	874	708,4		787	544	653	604	463	610,2	NC
msy-341540:5|chr2_517488_517505|	121	14	44	117	31	65,4		7	221	161	14	7	82	NC
Pmsy-341566:5|chr2_522559_522578[520957_523675]|	73	94	89	158	117	106,2		88	151	79	45	66	85,8	C
Pmsy-699656:3|chr2_536755_536770[536089_540804]|	42	59	24	65	58	49,6		61	59	46	84	50	60	C
Pmsy-341669:5|chr2_545820_545840[545568_547154]|	87	53	46	56	49	58,2		45	58	68	40	22	46,6	C
Pmsy-217859:3|chr2_564521_564537[563885_566842]|	155	92	159	165	136	141,4		74	213	162	105	138	138,4	C
Pmsy-46579:3|chr2_567207_567222[566886_567608]|	554	377	352	344	426	410,6		246	466	486	336	255	357,8	C
msy-479979:3|chr2_577472_577486|	62	57	92	109	85	81		56	121	61	40	90	73,6	NC

msy-147615:3|chr2_584590_584605|	272	150	156	238	132	189,6		65	234	280	120	118	163,4	NC
Pmsy-29354:5|chr2_661608_661626[661554_661858]|	3	1499	66	104	105	355,4		1019	95	2389	2200	80	1156,6	C
Pmsy-14522:3|chr2_676654_676671[676386_676674]|	1288	303	605	1315	257	753,6		234	481	642	711	847	583	C
Pmsy-14532:3|chr2_679299_679315[679161_680832]|	1264	482	507	1224	551	805,6		330	899	1414	1098	712	890,6	C
msy-14534:3|chr2_679301_679317|	5495	2488	2343	4473	2331	3426		1682	4048	5144	4834	2638	3669,2	NC
Pmsy-535732:5|chr2_685260_685277[682747_685822]|	138	0	1	1	101	48,2		0	1	190	103	127	84,2	C
Pmsy-218562:3|chr2_698300_698315[697668_699856]|	152	131	5	7	9	60,8		23	230	157	176	99	137	C
msy-47106:3|chr2_748283_748298|	367	271	247	477	162	304,8		214	376	342	185	197	262,8	NC
msy-218929:3|chr2_750953_750967|	130	91	144	201	96	132,4		65	143	158	63	146	115	NC
Pmsy-481646:3|chr2_801555_801571[801415_804038]|	99	75	0	108	0	56,4		67	160	119	0	1	69,4	C
msy-647127:5|chr2_831640_831660|	78	61	59	96	52	69,2		41	84	82	52	24	56,6	NC

msy-184347:5|chr2_843990_844011|	57	97	88	89	107	87,6		101	129	83	43	120	95,2	NC
msy-68743:5|chr2_856588_856609|	329	230	210	337	225	266,2		175	370	316	291	343	299	NC
Pmsy-14896:3|chr2_865136_865150[863823_865265]|	519	254	222	487	261	348,6		199	585	468	247	309	361,6	C
msy-148946:3|chr2_865138_865152|	4	429	2	4	342	156,2		0	915	632	309	319	435	NC
msy-702483:3|chr2_866111_866125|	394	8	133	10	12	111,4		10	23	7	12	11	12,6	NC
Pmsy-343228:5|chr2_866593_866615[866084_867254]|	51	35	29	69	34	43,6		28	77	54	62	43	52,8	C
Pmsy-385587:3|chr2_873775_873791[872748_875402]|	75	43	92	152	94	91,2		53	191	65	24	69	80,4	C
msy-149010:3|chr2_881321_881336|	96	371	234	402	129	246,4		267	184	104	43	136	146,8	NC
Pmsy-536941:5|chr2_889198_889218[887748_889397]|	36	40	0	6	177	51,8		98	0	107	104	92	80,2	C
Pmsy-343371:5|chr2_895626_895647[894826_896729]|	29	19	21	25	18	22,4		19	42	34	20	16	26,2	C
Pmsy-14994:3|chr2_927121_927136[926629_927267]|	75	65	100	30	49	63,8		26	82	81	63	42	58,8	C

msy-184750:5|chr2_935521_935541|	0	0	1	0	0	0,2		0	0	0	0	0	0	NC
Pmsy-3289:5|chr2_935521_935539[934899_937766]|	1925	981	1414	161	1112	1118,6		135	2451	1605	1093	1198	1296,4	C
Pmsy-149253:3|chr2_938237_938253[937796_939082]|	129	116	156	144	147	138,4		90	344	321	274	182	242,2	C
msy-15036:3|chr2_949070_949085|	1817	1019	2306	1219	1236	1519,4		670	1995	1807	1154	948	1314,8	NC
Pmsy-3299:5|chr2_952022_952042[951999_954019]|	537	7	5	8	391	189,6		206	469	12	395	20	220,4	C
msy-30050:5|chr2_984449_984468|	0	0	0	1	0	0,2		0	0	0	0	0	0	NC
Pmsy-3352:5|chr2_984449_984466[984381_984580]|	1839	786	1032	6	4	733,4		448	1414	1313	10	647	766,4	C
Pmsy-91552:3|chr2_985292_985307[985230_986147]|	239	188	124	203	266	204		145	284	242	226	162	211,8	C
msy-149478:3|chr2_986287_986303|	207	131	137	149	94	143,6		87	196	188	160	86	143,4	NC
msy-220302:3|chr2_1022326_1022341|	93	58	71	51	91	72,8		46	105	64	65	52	66,4	NC
Pmsy-15171:3|chr2_1022329_1022343[1021990_1024110]|	229	183	186	136	242	195,2		157	254	257	231	143	208,4	C

<table>
<tr><td>Pmsy-47827:3|chr2_1025816_1025831[1024303_1027074]|</td><td>1</td><td>2327</td><td>1490</td><td>6</td><td>2</td><td>765,2</td><td></td><td>2840</td><td>5</td><td>2730</td><td>5</td><td>3274</td><td>1770,8</td><td>C</td></tr>
<tr><td>Pmsy-386734:3|chr2_1050614_1050629[1050070_1050908]|</td><td>45</td><td>24</td><td>23</td><td>40</td><td>31</td><td>32,6</td><td></td><td>31</td><td>52</td><td>50</td><td>39</td><td>33</td><td>41</td><td>C</td></tr>
<tr><td>Pmsy-435711:5|chr2_1053401_1053420[1052457_1055279]|</td><td>172</td><td>81</td><td>83</td><td>151</td><td>47</td><td>106,8</td><td></td><td>37</td><td>1</td><td>106</td><td>1</td><td>0</td><td>29</td><td>C</td></tr>
<tr><td>msy-185271:5|chr2_1066784_1066803|</td><td>198</td><td>1</td><td>125</td><td>231</td><td>117</td><td>134,4</td><td></td><td>102</td><td>215</td><td>196</td><td>4</td><td>5</td><td>104,4</td><td>NC</td></tr>
<tr><td>msy-538060:5|chr2_1080246_1080267|</td><td>0</td><td>0</td><td>0</td><td>0</td><td>0</td><td>0</td><td></td><td>0</td><td>0</td><td>2</td><td>0</td><td>2</td><td>0,8</td><td>NC</td></tr>
<tr><td>msy-220733:3|chr2_1094103_1094118|</td><td>262</td><td>1</td><td>142</td><td>3</td><td>5</td><td>82,6</td><td></td><td>137</td><td>16</td><td>238</td><td>3</td><td>187</td><td>116,2</td><td>NC</td></tr>
<tr><td>msy-30302:5|chr2_1113611_1113628|</td><td>419</td><td>229</td><td>250</td><td>286</td><td>331</td><td>303</td><td></td><td>179</td><td>371</td><td>316</td><td>213</td><td>231</td><td>262</td><td>NC</td></tr>
<tr><td>Pmsy-119500:5|chr2_1115595_1115616[1114331_1119529]|</td><td>1</td><td>1</td><td>1</td><td>0</td><td>2</td><td>1</td><td></td><td>0</td><td>6</td><td>1</td><td>1</td><td>2</td><td>2</td><td>C</td></tr>
<tr><td>msy-3536:5|chr2_1120030_1120047|</td><td>624</td><td>539</td><td>841</td><td>603</td><td>898</td><td>701</td><td></td><td>333</td><td>952</td><td>960</td><td>423</td><td>679</td><td>669,4</td><td>NC</td></tr>
<tr><td>Pmsy-185473:5|chr2_1124027_1124046[1123994_1124570]|</td><td>127</td><td>82</td><td>101</td><td>89</td><td>103</td><td>100,4</td><td></td><td>145</td><td>165</td><td>132</td><td>304</td><td>106</td><td>170,4</td><td>C</td></tr>
<tr><td>msy-436304:5|chr2_1169815_1169835|</td><td>0</td><td>0</td><td>0</td><td>1</td><td>0</td><td>0,2</td><td></td><td>0</td><td>0</td><td>0</td><td>0</td><td>0</td><td>0</td><td>NC</td></tr>
</table>

<table>
<tr><td>Pmsy-69576:5|chr2_1169815_1169832[1167540_1171441]|</td><td>259</td><td>204</td><td>266</td><td>361</td><td>341</td><td>286,2</td><td></td><td>333</td><td>451</td><td>319</td><td>253</td><td>321</td><td>335,4</td><td>C</td></tr>
<tr><td>msy-185930:5|chr2_1232830_1232846|</td><td>230</td><td>1</td><td>181</td><td>174</td><td>151</td><td>147,4</td><td></td><td>92</td><td>237</td><td>196</td><td>0</td><td>0</td><td>105</td><td>NC</td></tr>
<tr><td>msy-484872:3|chr2_1237802_1237816|</td><td>29</td><td>30</td><td>16</td><td>36</td><td>30</td><td>28,2</td><td></td><td>39</td><td>37</td><td>37</td><td>35</td><td>25</td><td>34,6</td><td>NC</td></tr>
<tr><td>msy-3749:5|chr2_1262230_1262253|</td><td>0</td><td>2</td><td>3</td><td>6</td><td>7</td><td>3,6</td><td></td><td>2</td><td>2</td><td>1</td><td>2</td><td>2</td><td>1,8</td><td>NC</td></tr>
<tr><td>Pmsy-15703:3|chr2_1279717_1279732[1278825_1280582]|</td><td>2870</td><td>2654</td><td>2913</td><td>2982</td><td>3689</td><td>3021,6</td><td></td><td>2699</td><td>4627</td><td>3342</td><td>3366</td><td>3097</td><td>3426,2</td><td>C</td></tr>
<tr><td>Pmsy-48603:3|chr2_1290470_1290485[1290444_1290587]|</td><td>11</td><td>848</td><td>724</td><td>8</td><td>12</td><td>320,6</td><td></td><td>18</td><td>1295</td><td>759</td><td>15</td><td>12</td><td>419,8</td><td>C</td></tr>
<tr><td>msy-437025:5|chr2_1297434_1297451|</td><td>24</td><td>60</td><td>1</td><td>147</td><td>106</td><td>67,6</td><td></td><td>74</td><td>191</td><td>82</td><td>73</td><td>111</td><td>106,2</td><td>NC</td></tr>
<tr><td>msy-221885:3|chr2_1303819_1303833|</td><td>235</td><td>0</td><td>1</td><td>1</td><td>0</td><td>47,4</td><td></td><td>2</td><td>1</td><td>302</td><td>289</td><td>4</td><td>119,6</td><td>NC</td></tr>
<tr><td>msy-485332:3|chr2_1307001_1307016|</td><td>75</td><td>43</td><td>75</td><td>128</td><td>72</td><td>78,6</td><td></td><td>61</td><td>107</td><td>114</td><td>58</td><td>71</td><td>82,2</td><td>NC</td></tr>
<tr><td>msy-69971:5|chr2_1312011_1312031|</td><td>215</td><td>157</td><td>77</td><td>373</td><td>141</td><td>192,6</td><td></td><td>204</td><td>287</td><td>305</td><td>339</td><td>176</td><td>262,2</td><td>NC</td></tr>
<tr><td>msy-120218:5|chr2_1315910_1315929|</td><td>84</td><td>71</td><td>41</td><td>121</td><td>40</td><td>71,4</td><td></td><td>63</td><td>87</td><td>82</td><td>70</td><td>41</td><td>68,6</td><td>NC</td></tr>
</table>

msy-706910:3|chr2_1372300_1372315|	103	0	76	317	89	117		0	0	0	0	0	0	NC
msy-15888:3|chr3_1270_1286|	835	508	364	862	419	597,6		401	808	760	508	331	561,6	NC
msy-15908:3|chr3_14235_14251|	553	221	291	470	206	348,2		107	434	600	230	170	308,2	NC
Pmsy-30877:5|chr3_18988_19007[18234_21461]|	397	265	166	352	221	280,2		206	436	397	299	255	318,6	C
Pmsy-49009:3|chr3_60150_60165[54537_61004]|	389	187	150	254	128	221,6		96	258	262	147	91	170,8	C
msy-70294:5|chr3_67755_67774|	4	429	253	1	3	138		480	573	1	5	0	211,8	NC
msy-120663:5|chr3_74777_74800|	1	0	0	0	0	0,2		0	0	0	0	0	0	NC
msy-120865:5|chr3_133550_133572|	16	15	22	16	28	19,4		18	42	28	21	30	27,8	NC
msy-389810:3|chr3_135647_135662|	154	65	146	104	76	109		20	93	82	46	48	57,8	NC
msy-346333:5|chr3_136032_136049|	146	82	191	114	17	110		5	23	143	7	5	36,6	NC
msy-152035:3|chr3_139089_139103|	116	1	0	136	78	66,2		88	0	119	0	2	41,8	NC
msy-70533:5|chr3_166029_166051|	6	4	2	3	3	3,6		4	8	9	4	3	5,6	NC

msy-70551:5|chr3_172264_172283|	252	250	185	539	189	283		193	376	346	213	218	269,2	NC
msy-93632:3|chr3_183622_183638|	65	213	280	97	229	176,8		271	204	130	44	65	142,8	NC
msy-49355:3|chr3_185081_185097|	532	244	362	456	209	360,6		110	360	459	228	182	267,8	NC
msy-187359:5|chr3_217906_217926|	122	65	134	109	93	104,6		52	136	123	68	90	93,8	NC
Pmsy-346778:5|chr3_226337_226357[226074_226670]|	28	25	25	34	32	28,8		27	41	41	23	27	31,8	C
Pmsy-152753:3|chr3_306814_306828[305998_307536]|	219	151	191	308	302	234,2		140	374	257	161	380	262,4	C
msy-488182:3|chr3_324055_324070|	7	128	100	187	10	86,4		4	139	6	120	12	56,2	NC
Pmsy-652564:5|chr3_334514_334536[333582_334941]|	0	0	1	0	1	0,4		1	0	0	0	1	0,4	C
Pmsy-263921:5|chr3_386613_386631[385273_386799]|	120	80	163	130	112	121		47	176	116	60	102	100,2	C
msy-4413:5|chr3_398639_398660|	738	509	250	970	641	621,6		616	909	647	1380	818	874	NC
Pmsy-121930:5|chr3_423042_423066[422853_428156]|	0	1	0	1	0	0,4		0	0	0	0	0	0	C

msy-16684:3|chr3_435408_435427|	3321	2619	3812	3566	4321	3527,8		2581	5001	2743	2005	3956	3257,2 NC
msy-224680:3|chr3_435408_435422|	0	0	0	0	1	0,2		0	0	2	0	0	0,4 NC
msy-153439:3|chr3_437708_437722|	139	105	185	145	220	158,8		97	317	102	72	264	170,4 NC
msy-224868:3|chr3_467688_467703|	83	127	122	1	1	66,8		145	125	68	38	1	75,4 NC
Pmsy-16766:3|chr3_482926_482940[482027_483883]|	563	385	462	605	383	479,6		243	501	481	178	290	338,6 C
msy-188573:5|chr3_493700_493721|	15	25	18	8	14	16		5	28	13	16	18	16 NC
msy-4580:5|chr3_503438_503459|	6	3	6	5	8	5,6		9	4	9	2	3	5,4 NC
msy-153746:3|chr3_504566_504581|	106	142	268	120	84	144		105	355	248	77	166	190,2 NC
Pmsy-225140:3|chr3_523255_523271[523014_523892]|	138	97	127	116	132	122		84	142	136	111	93	113,2 C
msy-4613:5|chr3_529378_529397|	11388	10928	7434	19070	11836	12131,2		15764	17130	14143	16440	11242	14943,8 NC
Pmsy-16885:3|chr3_549890_549906[548902_550441]|	2182	1391	2482	1557	1682	1858,8		974	2678	2397	1797	1532	1875,6 C
msy-16887:3|chr3_549892_549907|	267	205	279	193	188	226,4		95	319	313	354	158	247,8 NC

Pmsy-32018:5|chr3_568741_568763[567336_569195]|	279	191	245	335	166	243,2		140	361	312	183	253	249,8	C
msy-16921:3|chr3_569271_569287|	4784	5443	4280	3906	3832	4449		8831	4962	3783	2692	2676	4588,8	NC
msy-597152:3|chr3_595827_595842|	88	61	44	71	77	68,2		68	114	102	132	94	102	NC
msy-122580:5|chr3_603779_603796|	173	146	219	232	191	192,2		100	349	195	93	193	186	NC
msy-490489:3|chr3_606049_606063|	5	7	156	112	122	80,4		75	143	0	114	90	84,4	NC
Pmsy-348769:5|chr3_612862_612882[610592_613036]|	39	48	94	61	63	61		35	84	76	52	54	60,2	C
Pmsy-225706:3|chr3_626128_626143[624932_626465]|	0	16	0	125	80	44,2		90	356	149	16	146	151,4	C
Pmsy-225845:3|chr3_650560_650574[650342_652917]|	380	7	10	258	478	226,6		7	14	503	651	363	307,6	C
msy-225871:3|chr3_654381_654395|	216	96	122	169	87	138		33	143	196	133	92	119,4	NC
msy-712812:3|chr3_654383_654397|	73	49	43	62	34	52,2		36	67	47	34	33	43,4	NC
Pmsy-71903:5|chr3_663218_663240[662492_663445]|	283	3	4	606	8	180,8		596	535	519	344	10	400,8	C

<table>
<tr><th></th><th></th><th></th><th></th><th></th><th></th><th></th><th></th><th></th><th></th><th></th><th></th><th></th><th></th><th></th></tr>
<tr><td>Pmsy-305780:3|chr3_664601_664617[664545_664696]|</td><td>262</td><td>175</td><td>260</td><td>249</td><td>106</td><td>210,4</td><td></td><td>57</td><td>411</td><td>192</td><td>112</td><td>158</td><td>186</td><td>C</td></tr>
<tr><td>msy-95283:3|chr3_668339_668353|</td><td>9</td><td>11</td><td>1</td><td>22</td><td>14</td><td>11,4</td><td></td><td>20</td><td>14</td><td>14</td><td>36</td><td>15</td><td>19,8</td><td>NC</td></tr>
<tr><td>Pmsy-122863:5|chr3_679906_679925[678941_682138]|</td><td>1</td><td>1</td><td>0</td><td>0</td><td>3</td><td>1</td><td></td><td>0</td><td>0</td><td>2</td><td>0</td><td>2</td><td>0,8</td><td>C</td></tr>
<tr><td>msy-154712:3|chr3_710546_710561|</td><td>62</td><td>420</td><td>38</td><td>26</td><td>585</td><td>226,2</td><td></td><td>22</td><td>760</td><td>726</td><td>437</td><td>34</td><td>395,8</td><td>NC</td></tr>
<tr><td>msy-544155:5|chr3_715483_715500|</td><td>122</td><td>84</td><td>58</td><td>15</td><td>103</td><td>76,4</td><td></td><td>72</td><td>7</td><td>136</td><td>8</td><td>64</td><td>57,4</td><td>NC</td></tr>
<tr><td>msy-349457:5|chr3_744711_744731|</td><td>0</td><td>0</td><td>1</td><td>0</td><td>0</td><td>0,2</td><td></td><td>0</td><td>0</td><td>0</td><td>0</td><td>0</td><td>0</td><td>NC</td></tr>
<tr><td>Pmsy-72123:5|chr3_744711_744729[744309_745742]|</td><td>314</td><td>19</td><td>132</td><td>46</td><td>83</td><td>118,8</td><td></td><td>238</td><td>41</td><td>311</td><td>27</td><td>215</td><td>166,4</td><td>C</td></tr>
<tr><td>msy-491686:3|chr3_772457_772471|</td><td>14</td><td>10</td><td>5</td><td>22</td><td>47</td><td>19,6</td><td></td><td>38</td><td>100</td><td>17</td><td>11</td><td>77</td><td>48,6</td><td>NC</td></tr>
<tr><td>Pmsy-598695:3|chr3_796981_796995[795659_797629]|</td><td>46</td><td>29</td><td>43</td><td>38</td><td>38</td><td>38,8</td><td></td><td>22</td><td>68</td><td>43</td><td>32</td><td>42</td><td>41,4</td><td>C</td></tr>
<tr><td>Pmsy-598742:3|chr3_801809_801823[800378_804013]|</td><td>83</td><td>33</td><td>57</td><td>57</td><td>49</td><td>55,8</td><td></td><td>37</td><td>67</td><td>76</td><td>101</td><td>67</td><td>69,6</td><td>C</td></tr>
<tr><td>msy-394332:3|chr3_811251_811266|</td><td>11</td><td>15</td><td>32</td><td>8</td><td>16</td><td>16,4</td><td></td><td>20</td><td>14</td><td>7</td><td>5</td><td>12</td><td>11,6</td><td>NC</td></tr>
</table>

msy-123405:5|chr3_820504_820523|	177	103	145	201	199	165		140	314	173	253	234	222,8	NC
msy-5034:5|chr3_831309_831328|	616	452	209	841	211	465,8		303	947	760	663	200	574,6	NC
msy-32607:5|chr3_831309_831330|	0	1	0	0	2	0,6		0	0	0	0	0	0	NC
Pmsy-155405:3|chr3_864587_864602[863744_865027]|	259	149	286	247	178	223,8		119	383	273	162	184	224,2	C
msy-599336:3|chr3_876539_876555|	0	78	103	1	1	36,6		1	202	1	52	104	72	NC
Pmsy-190278:5|chr3_903153_903172[901078_905277]|	122	100	110	88	153	114,6		81	211	158	97	148	139	C
msy-442499:5|chr3_903565_903580|	60	67	120	28	109	76,8		75	114	75	6	31	60,2	NC
msy-17522:3|chr3_910480_910495|	889	688	542	853	810	756,4		499	1086	1294	1221	684	956,8	NC
msy-492772:3|chr3_917914_917929|	47	80	53	35	82	59,4		62	117	85	98	67	85,8	NC
msy-599814:3|chr3_930272_930286|	113	69	70	80	89	84,2		54	105	139	63	64	85	NC
msy-307483:3|chr3_948924_948938|	1	2	0	0	5	1,6		1	12	0	5	4	4,4	NC

<table>
<tr><td>Pmsy-227468:3|chr3_948925_948940[947004_950558]|</td><td>233</td><td>142</td><td>120</td><td>199</td><td>121</td><td>163</td><td></td><td>102</td><td>186</td><td>248</td><td>188</td><td>123</td><td>169,4</td><td>C</td></tr>
<tr><td>msy-17650:3|chr3_973226_973241|</td><td>816</td><td>394</td><td>397</td><td>656</td><td>304</td><td>513,4</td><td></td><td>251</td><td>591</td><td>771</td><td>503</td><td>311</td><td>485,4</td><td>NC</td></tr>
<tr><td>msy-5211:5|chr3_978180_978201|</td><td>174</td><td>45</td><td>83</td><td>100</td><td>170</td><td>114,4</td><td></td><td>55</td><td>249</td><td>225</td><td>85</td><td>78</td><td>138,4</td><td>NC</td></tr>
<tr><td>Pmsy-155971:3|chr3_982764_982780[982575_982803]|</td><td>269</td><td>154</td><td>172</td><td>363</td><td>214</td><td>234,4</td><td></td><td>4</td><td>420</td><td>284</td><td>152</td><td>9</td><td>173,8</td><td>C</td></tr>
<tr><td>msy-124034:5|chr3_1010282_1010302|</td><td>139</td><td>149</td><td>99</td><td>130</td><td>246</td><td>152,6</td><td></td><td>178</td><td>205</td><td>125</td><td>288</td><td>145</td><td>188,2</td><td>NC</td></tr>
<tr><td>msy-266820:5|chr3_1014947_1014968|</td><td>1</td><td>0</td><td>1</td><td>1</td><td>0</td><td>0,6</td><td></td><td>3</td><td>3</td><td>0</td><td>0</td><td>4</td><td>2</td><td>NC</td></tr>
<tr><td>msy-72864:5|chr3_1035753_1035769|</td><td>234</td><td>186</td><td>199</td><td>156</td><td>166</td><td>188,2</td><td></td><td>201</td><td>183</td><td>178</td><td>128</td><td>96</td><td>157,2</td><td>NC</td></tr>
<tr><td>msy-51650:3|chr3_1039340_1039354|</td><td>260</td><td>241</td><td>184</td><td>511</td><td>240</td><td>287,2</td><td></td><td>185</td><td>480</td><td>324</td><td>242</td><td>294</td><td>305</td><td>NC</td></tr>
<tr><td>msy-5320:5|chr3_1050654_1050670|</td><td>1208</td><td>669</td><td>858</td><td>893</td><td>815</td><td>888,6</td><td></td><td>561</td><td>967</td><td>951</td><td>521</td><td>505</td><td>701</td><td>NC</td></tr>
<tr><td>Pmsy-5338:5|chr3_1065207_1065228[1063502_1066888]|</td><td>340</td><td>13</td><td>17</td><td>303</td><td>289</td><td>192,4</td><td></td><td>15</td><td>49</td><td>15</td><td>9</td><td>42</td><td>26</td><td>C</td></tr>
<tr><td>msy-33075:5|chr3_1071082_1071100|</td><td>487</td><td>284</td><td>396</td><td>465</td><td>335</td><td>393,4</td><td></td><td>166</td><td>530</td><td>541</td><td>233</td><td>311</td><td>356,2</td><td>NC</td></tr>
</table>

msy-267189:5|chr3_1094501_1094519|	107	92	99	180	118	119,2		66	183	107	50	100	101,2	NC
Pmsy-716753:3|chr3_1111794_1111809[1108728_1112546]|	89	81	2	92	110	74,8		1	0	5	99	117	44,4	C
msy-124448:5|chr3_1132393_1132418|	0	0	1	0	0	0,2		0	0	0	0	0	0	NC
Pmsy-546603:5|chr3_1148194_1148212[1148138_1148393]|	57	50	36	102	50	59		53	134	76	67	71	80,2	C
Pmsy-51952:3|chr3_1152653_1152667[1152024_1156436]|	10	2	4	67	8	18,2		98	11	3	6	122	48	C
msy-96944:3|chr3_1152654_1152669|	502	732	295	968	1328	765		1347	1145	677	1187	1665	1204,2	NC
msy-267434:5|chr3_1154898_1154914|	2	86	0	158	3	49,8		125	168	2	156	135	117,2	NC
msy-5457:5|chr3_1158310_1158330|	2031	2179	2847	2912	2646	2523		2697	5713	2960	1579	3142	3218,2	NC
msy-33251:5|chr3_1158310_1158332|	0	0	0	0	0	0		0	0	0	1	0	0,2	NC
msy-494561:3|chr3_1163289_1163305|	37	35	16	50	101	47,8		80	284	37	33	113	109,4	NC
Pmsy-601818:3|chr3_1177820_1177834[1177671_1177900]|	78	1	120	1	0	40		134	1	113	0	92	68	C

msy-228879:3|chr3_1209787_1209803|	104	181	86	5	200	115,2		2	377	212	307	151	209,8	NC
Pmsy-52130:3|chr3_1217562_1217578[1217195_1217778]|	413	380	200	608	334	387		229	668	581	301	358	427,4	C
msy-52152:3|chr3_1224636_1224650|	190	118	184	198	136	165,2		112	272	161	88	129	152,4	NC
Pmsy-547250:5|chr3_1255666_1255687[1254946_1257936]|	34	19	13	34	26	25,2		14	60	31	20	26	30,2	C
Pmsy-658820:5|chr3_1294466_1294481[1294380_1295050]|	34	92	66	36	72	60		76	34	47	47	64	53,6	C
Pmsy-18215:3|chr3_1294821_1294835[1294785_1295424]|	382	233	514	335	420	376,8		216	594	370	263	486	385,8	C
msy-125046:5|chr3_1297084_1297102|	40	43	981	503	47	322,8		380	120	899	55	584	407,6	NC
Pmsy-5674:5|chr3_1318523_1318542[1316425_1319358]|	314	232	130	334	170	236		97	279	315	259	161	222,2	C
Pmsy-125144:5|chr3_1322488_1322503[1320505_1323678]|	0	247	0	0	0	49,4		0	320	210	117	1	129,6	C
Pmsy-5695:5|chr3_1332843_1332863[1331184_1333122]|	1014	555	311	8	608	499,2		3	693	747	492	592	505,4	C
msy-309914:3|chr3_1340918_1340942|	99	100	123	107	117	109,2		111	151	106	103	100	114,2	NC

<table>
<tr><td>Pmsy-496124:3|chr4_18845_18861[18106_20873]|</td><td>65</td><td>61</td><td>59</td><td>77</td><td>80</td><td>68,4</td><td></td><td>50</td><td>122</td><td>75</td><td>91</td><td>96</td><td>86,8</td><td>C</td></tr>
<tr><td>msy-97721:3|chr4_22409_22424|</td><td>322</td><td>34</td><td>1814</td><td>150</td><td>60</td><td>476</td><td></td><td>1198</td><td>3067</td><td>59</td><td>162</td><td>2111</td><td>1319,4</td><td>NC</td></tr>
<tr><td>Pmsy-157878:3|chr4_30210_30225[29036_31384]|</td><td>240</td><td>123</td><td>173</td><td>175</td><td>107</td><td>163,6</td><td></td><td>95</td><td>266</td><td>220</td><td>121</td><td>100</td><td>160,4</td><td>C</td></tr>
<tr><td>msy-73768:5|chr4_40171_40195|</td><td>15</td><td>20</td><td>28</td><td>20</td><td>22</td><td>21</td><td></td><td>22</td><td>28</td><td>25</td><td>15</td><td>14</td><td>20,8</td><td>NC</td></tr>
<tr><td>Pmsy-18469:3|chr4_85179_85194[84770_85663]|</td><td>846</td><td>500</td><td>568</td><td>573</td><td>798</td><td>657</td><td></td><td>637</td><td>1221</td><td>692</td><td>788</td><td>752</td><td>818</td><td>C</td></tr>
<tr><td>Pmsy-268754:5|chr4_92315_92333[92249_92674]|</td><td>6</td><td>6</td><td>89</td><td>400</td><td>19</td><td>104</td><td></td><td>238</td><td>308</td><td>15</td><td>99</td><td>182</td><td>168,4</td><td>C</td></tr>
<tr><td>Pmsy-310485:3|chr4_95442_95456[93124_97499]|</td><td>125</td><td>109</td><td>97</td><td>90</td><td>41</td><td>92,4</td><td></td><td>47</td><td>161</td><td>141</td><td>54</td><td>41</td><td>88,8</td><td>C</td></tr>
<tr><td>msy-398656:3|chr4_108836_108850|</td><td>37</td><td>19</td><td>27</td><td>29</td><td>35</td><td>29,4</td><td></td><td>21</td><td>43</td><td>37</td><td>89</td><td>37</td><td>45,4</td><td>NC</td></tr>
<tr><td>msy-125676:5|chr4_118735_118755|</td><td>3</td><td>3</td><td>1</td><td>4</td><td>0</td><td>2,2</td><td></td><td>1</td><td>2</td><td>0</td><td>0</td><td>0</td><td>0,6</td><td>NC</td></tr>
<tr><td>Pmsy-33911:5|chr4_127835_127853[127775_127997]|</td><td>331</td><td>231</td><td>195</td><td>229</td><td>261</td><td>249,4</td><td></td><td>229</td><td>389</td><td>298</td><td>329</td><td>217</td><td>292,4</td><td>C</td></tr>
<tr><td>Pmsy-158381:3|chr4_132204_132219[130619_132872]|</td><td>270</td><td>143</td><td>181</td><td>216</td><td>113</td><td>184,6</td><td></td><td>117</td><td>318</td><td>244</td><td>158</td><td>157</td><td>198,8</td><td>C</td></tr>
<tr><td>Pmsy-398952:3|chr4_150249_150264[146401_150705]|</td><td>96</td><td>53</td><td>98</td><td>83</td><td>121</td><td>90,2</td><td></td><td>54</td><td>180</td><td>71</td><td>55</td><td>96</td><td>91,2</td><td>C</td></tr>
</table>

<table>
<tr><td>msy-33957:5|chr4_151332_151350|</td><td>243</td><td>316</td><td>732</td><td>203</td><td>454</td><td>389,6</td><td></td><td>356</td><td>446</td><td>356</td><td>224</td><td>356</td><td>347,6</td><td>NC</td></tr>
<tr><td>msy-269268:5|chr4_207657_207679|</td><td>2</td><td>0</td><td>0</td><td>1</td><td>0</td><td>0,6</td><td></td><td>0</td><td>1</td><td>0</td><td>0</td><td>0</td><td>0,2</td><td>NC</td></tr>
<tr><td>Pmsy-497749:3|chr4_239835_239851[238928_240945]|</td><td>51</td><td>25</td><td>97</td><td>52</td><td>44</td><td>53,8</td><td></td><td>35</td><td>73</td><td>56</td><td>33</td><td>46</td><td>48,6</td><td>C</td></tr>
<tr><td>msy-311348:3|chr4_242708_242723|</td><td>9</td><td>7</td><td>7</td><td>954</td><td>720</td><td>339,4</td><td></td><td>4</td><td>9</td><td>14</td><td>1019</td><td>3</td><td>209,8</td><td>NC</td></tr>
<tr><td>Pmsy-193103:5|chr4_247654_247675[247068_249497]|</td><td>88</td><td>36</td><td>21</td><td>90</td><td>64</td><td>59,8</td><td></td><td>45</td><td>80</td><td>76</td><td>97</td><td>82</td><td>76</td><td>C</td></tr>
<tr><td>Pmsy-126197:5|chr4_266775_266794[263835_267104]|</td><td>0</td><td>0</td><td>0</td><td>0</td><td>1</td><td>0,2</td><td></td><td>1</td><td>0</td><td>0</td><td>0</td><td>0</td><td>0,2</td><td>C</td></tr>
<tr><td>msy-721245:3|chr4_271848_271863|</td><td>59</td><td>45</td><td>30</td><td>90</td><td>31</td><td>51</td><td></td><td>31</td><td>97</td><td>81</td><td>43</td><td>61</td><td>62,6</td><td>NC</td></tr>
<tr><td>Pmsy-498019:3|chr4_273175_273191[273061_274953]|</td><td>32</td><td>77</td><td>119</td><td>114</td><td>103</td><td>89</td><td></td><td>73</td><td>154</td><td>104</td><td>53</td><td>124</td><td>101,6</td><td>C</td></tr>
<tr><td>msy-6088:5|chr4_273774_273790|</td><td>862</td><td>5</td><td>3</td><td>942</td><td>1120</td><td>586,4</td><td></td><td>1077</td><td>13</td><td>8</td><td>1203</td><td>1107</td><td>681,6</td><td>NC</td></tr>
<tr><td>Pmsy-159062:3|chr4_283069_283085[282788_288334]|</td><td>335</td><td>143</td><td>206</td><td>351</td><td>150</td><td>237</td><td></td><td>89</td><td>277</td><td>314</td><td>156</td><td>146</td><td>196,4</td><td>C</td></tr>
<tr><td>msy-193246:5|chr4_286267_286287|</td><td>10</td><td>5</td><td>246</td><td>260</td><td>406</td><td>185,4</td><td></td><td>344</td><td>9</td><td>393</td><td>277</td><td>5</td><td>205,6</td><td>NC</td></tr>
</table>

msy-498137:3|chr4_288001_288015|	97	58	49	96	30	66		28	83	97	47	33	57,6	NC
Pmsy-18847:3|chr4_290001_290017[289373_291790]|	1226	730	625	9	8	519,6		6	16	14	628	7	134,2	C
msy-18885:3|chr4_310762_310776|	801	86	89	784	218	395,6		343	1236	1034	687	610	782	NC
msy-126365:5|chr4_316262_316278|	218	156	251	209	162	199,2		110	323	257	162	162	202,8	NC
msy-269760:5|chr4_317995_318015|	0	0	0	1	0	0,2		0	0	0	1	0	0,2	NC
msy-126367:5|chr4_317996_318013|	106	134	186	52	152	126		199	93	77	70	49	97,6	NC
msy-231407:3|chr4_320632_320646|	162	20	105	167	107	112,2		66	161	158	21	25	86,2	NC
Pmsy-6157:5|chr4_322467_322487[322435_322713]|	668	428	468	794	522	576		478	1031	645	427	513	618,8	C
Pmsy-606051:3|chr4_345383_345397[344326_346210]|	0	25	0	0	1	5,2		29	1	3	20	16	13,8	C
msy-98898:3|chr4_350694_350709|	179	179	160	223	103	168,8		164	201	136	51	112	132,8	NC
Pmsy-312182:3|chr4_371492_371508[370538_373505]|	148	74	69	120	52	92,6		32	151	106	70	55	82,8	C

<table>
<tr><th></th><th></th><th></th><th></th><th></th><th></th><th></th><th></th><th></th><th></th><th></th><th></th><th></th><th></th><th></th></tr>
<tr><td>Pmsy-126590:5|chr4_387997_388014[387764_388124]|</td><td>115</td><td>130</td><td>150</td><td>162</td><td>195</td><td>150,4</td><td></td><td>188</td><td>183</td><td>128</td><td>150</td><td>180</td><td>165,8</td><td>C</td></tr>
<tr><td>Pmsy-312321:3|chr4_392450_392466[392342_394034]|</td><td>65</td><td>67</td><td>55</td><td>118</td><td>74</td><td>75,8</td><td></td><td>72</td><td>202</td><td>86</td><td>65</td><td>95</td><td>104</td><td>C</td></tr>
<tr><td>msy-74715:5|chr4_408923_408944|</td><td>68</td><td>36</td><td>73</td><td>75</td><td>43</td><td>59</td><td></td><td>19</td><td>57</td><td>44</td><td>34</td><td>35</td><td>37,8</td><td>NC</td></tr>
<tr><td>msy-550429:5|chr4_451480_451503|</td><td>0</td><td>0</td><td>1</td><td>0</td><td>0</td><td>0,2</td><td></td><td>0</td><td>0</td><td>0</td><td>0</td><td>0</td><td>0</td><td>NC</td></tr>
<tr><td>Pmsy-354620:5|chr4_456737_456754[456624_458729]|</td><td>4</td><td>0</td><td>195</td><td>0</td><td>3</td><td>40,4</td><td></td><td>80</td><td>3</td><td>207</td><td>179</td><td>115</td><td>116,8</td><td>C</td></tr>
<tr><td>msy-53869:3|chr4_514247_514262|</td><td>325</td><td>236</td><td>318</td><td>385</td><td>203</td><td>293,4</td><td></td><td>68</td><td>335</td><td>440</td><td>226</td><td>126</td><td>239</td><td>NC</td></tr>
<tr><td>msy-270700:5|chr4_520983_521001|</td><td>57</td><td>108</td><td>124</td><td>74</td><td>128</td><td>98,2</td><td></td><td>192</td><td>122</td><td>66</td><td>59</td><td>78</td><td>103,4</td><td>NC</td></tr>
<tr><td>msy-75164:5|chr4_575796_575813|</td><td>257</td><td>192</td><td>137</td><td>280</td><td>267</td><td>226,6</td><td></td><td>253</td><td>541</td><td>235</td><td>294</td><td>313</td><td>327,2</td><td>NC</td></tr>
<tr><td>Pmsy-19371:3|chr4_597407_597422[594514_598054]|</td><td>1300</td><td>2484</td><td>2800</td><td>4440</td><td>3124</td><td>2829,6</td><td></td><td>2696</td><td>6858</td><td>2933</td><td>1303</td><td>3530</td><td>3464</td><td>C</td></tr>
<tr><td>msy-724208:3|chr4_610831_610846|</td><td>200</td><td>0</td><td>0</td><td>3</td><td>78</td><td>56,2</td><td></td><td>66</td><td>0</td><td>165</td><td>0</td><td>65</td><td>59,2</td><td>NC</td></tr>
<tr><td>msy-402002:3|chr4_614385_614399|</td><td>84</td><td>48</td><td>112</td><td>109</td><td>84</td><td>87,4</td><td></td><td>41</td><td>122</td><td>104</td><td>71</td><td>76</td><td>82,8</td><td>NC</td></tr>
</table>

<table>
<tr><td></td><td></td><td></td><td></td><td></td><td></td><td></td><td></td><td></td><td></td><td></td><td></td><td></td><td></td><td></td></tr>
<tr><td>Pmsy-448319:5|chr4_615747_615764[615550_615810]|</td><td>94</td><td>80</td><td>96</td><td>78</td><td>0</td><td>69,6</td><td></td><td>58</td><td>160</td><td>0</td><td>0</td><td>65</td><td>56,6</td><td>C</td></tr>
<tr><td>msy-355679:5|chr4_663370_663391|</td><td>112</td><td>76</td><td>137</td><td>102</td><td>59</td><td>97,2</td><td></td><td>63</td><td>90</td><td>68</td><td>55</td><td>57</td><td>66,6</td><td>NC</td></tr>
<tr><td>Pmsy-271357:5|chr4_668018_668036[667100_670318]|</td><td>118</td><td>56</td><td>62</td><td>129</td><td>76</td><td>88,2</td><td></td><td>56</td><td>154</td><td>110</td><td>68</td><td>100</td><td>97,6</td><td>C</td></tr>
<tr><td>Pmsy-314062:3|chr4_690951_690966[690511_691496]|</td><td>82</td><td>76</td><td>60</td><td>72</td><td>121</td><td>82,2</td><td></td><td>94</td><td>123</td><td>133</td><td>149</td><td>108</td><td>121,4</td><td>C</td></tr>
<tr><td>msy-194933:5|chr4_699257_699276|</td><td>233</td><td>0</td><td>3</td><td>116</td><td>0</td><td>70,4</td><td></td><td>7</td><td>243</td><td>270</td><td>3</td><td>113</td><td>127,2</td><td>NC</td></tr>
<tr><td>msy-54426:3|chr4_710148_710162|</td><td>139</td><td>409</td><td>334</td><td>106</td><td>394</td><td>276,4</td><td></td><td>400</td><td>229</td><td>172</td><td>109</td><td>170</td><td>216</td><td>NC</td></tr>
<tr><td>msy-161117:3|chr4_711026_711040|</td><td>0</td><td>2</td><td>0</td><td>0</td><td>1</td><td>0,6</td><td></td><td>0</td><td>1</td><td>0</td><td>0</td><td>0</td><td>0,2</td><td>NC</td></tr>
<tr><td>msy-100138:3|chr4_711027_711043|</td><td>304</td><td>377</td><td>445</td><td>941</td><td>455</td><td>504,4</td><td></td><td>506</td><td>1183</td><td>729</td><td>377</td><td>557</td><td>670,4</td><td>NC</td></tr>
<tr><td>Pmsy-19670:3|chr4_751749_751764[751301_752392]|</td><td>1440</td><td>1283</td><td>850</td><td>1659</td><td>1654</td><td>1377,2</td><td></td><td>1361</td><td>2234</td><td>2099</td><td>2401</td><td>1556</td><td>1930,2</td><td>C</td></tr>
<tr><td>Pmsy-449094:5|chr4_762587_762602[762403_762735]|</td><td>83</td><td>74</td><td>139</td><td>31</td><td>55</td><td>76,4</td><td></td><td>44</td><td>75</td><td>65</td><td>36</td><td>39</td><td>51,8</td><td>C</td></tr>
<tr><td>msy-552446:5|chr4_783874_783896|</td><td>2</td><td>2</td><td>2</td><td>1</td><td>1</td><td>1,6</td><td></td><td>1</td><td>1</td><td>1</td><td>1</td><td>2</td><td>1,2</td><td>NC</td></tr>
</table>

<table>
<tr><td>msy-19745:3|chr4_789212_789227|</td><td>1676</td><td>2854</td><td>4696</td><td>1122</td><td>3974</td><td>2864,4</td><td></td><td>2435</td><td>3489</td><td>2215</td><td>415</td><td>975</td><td>1905,8</td><td>NC</td></tr>
<tr><td>Pmsy-19818:3|chr4_833995_834010[833817_834181]|</td><td>46</td><td>26</td><td>54</td><td>37</td><td>41</td><td>40,8</td><td></td><td>45</td><td>43</td><td>28</td><td>28</td><td>23</td><td>33,4</td><td>C</td></tr>
<tr><td>msy-128320:5|chr4_880442_880462|</td><td>97</td><td>60</td><td>70</td><td>95</td><td>107</td><td>85,8</td><td></td><td>70</td><td>185</td><td>123</td><td>130</td><td>119</td><td>125,4</td><td>NC</td></tr>
<tr><td>Pmsy-128398:5|chr4_907584_907605[906785_908273]|</td><td>27</td><td>25</td><td>30</td><td>32</td><td>28</td><td>28,4</td><td></td><td>25</td><td>64</td><td>27</td><td>29</td><td>31</td><td>35,2</td><td>C</td></tr>
<tr><td>msy-6902:5|chr4_922922_922943|</td><td>730</td><td>381</td><td>1155</td><td>501</td><td>621</td><td>677,6</td><td></td><td>253</td><td>1121</td><td>816</td><td>502</td><td>572</td><td>652,8</td><td>NC</td></tr>
<tr><td>msy-553237:5|chr4_928354_928375|</td><td>30</td><td>28</td><td>32</td><td>38</td><td>48</td><td>35,2</td><td></td><td>26</td><td>49</td><td>30</td><td>12</td><td>25</td><td>28,4</td><td>NC</td></tr>
<tr><td>Pmsy-6950:5|chr4_960202_960222[959315_960376]|</td><td>1459</td><td>1096</td><td>820</td><td>1746</td><td>1001</td><td>1224,4</td><td></td><td>801</td><td>1620</td><td>1239</td><td>675</td><td>1176</td><td>1102,2</td><td>C</td></tr>
<tr><td>msy-128573:5|chr4_961351_961372|</td><td>1</td><td>1</td><td>2</td><td>0</td><td>3</td><td>1,4</td><td></td><td>2</td><td>1</td><td>1</td><td>2</td><td>1</td><td>1,4</td><td>NC</td></tr>
<tr><td>Pmsy-76106:5|chr4_969394_969414[969021_969979]|</td><td>166</td><td>227</td><td>253</td><td>498</td><td>493</td><td>327,4</td><td></td><td>392</td><td>457</td><td>340</td><td>315</td><td>599</td><td>420,6</td><td>C</td></tr>
<tr><td>msy-6972:5|chr4_977808_977828|</td><td>0</td><td>0</td><td>1</td><td>0</td><td>0</td><td>0,2</td><td></td><td>1</td><td>2</td><td>1</td><td>0</td><td>0</td><td>0,8</td><td>NC</td></tr>
<tr><td>msy-235007:3|chr4_986158_986172|</td><td>176</td><td>101</td><td>87</td><td>78</td><td>61</td><td>100,6</td><td></td><td>32</td><td>181</td><td>102</td><td>54</td><td>79</td><td>89,6</td><td>NC</td></tr>
<tr><td>msy-35612:5|chr4_999849_999868|</td><td>473</td><td>196</td><td>247</td><td>183</td><td>607</td><td>341,2</td><td></td><td>223</td><td>335</td><td>482</td><td>534</td><td>473</td><td>409,4</td><td>NC</td></tr>
</table>

msy-553651:5|chr4_999849_999870|	0	0	0	0	1	0,2		0	0	0	0	1	0,2	NC
msy-20117:3|chr4_1007322_1007337|	678	841	982	1369	968	967,6		828	1761	1199	850	963	1120,2	NC
msy-162464:3|chr4_1007324_1007338|	385	449	563	711	547	531		502	875	556	455	551	587,8	NC
msy-20136:3|chr4_1018789_1018804|	1242	771	862	1043	1349	1053,4		662	1737	1471	1255	1318	1288,6	NC
Pmsy-76246:5|chr4_1019143_1019164[1016102_1020778]|	13	380	7	16	388	160,8		310	706	1064	849	29	591,6	C
Pmsy-128807:5|chr4_1024162_1024180[1023781_1026396]|	254	113	259	209	165	200		65	283	304	88	141	176,2	C
msy-162592:3|chr4_1034129_1034144|	99	88	0	133	1	64,2		1	814	0	369	306	298	NC
Pmsy-450567:5|chr4_1043581_1043598[1040884_1044360]|	41	85	57	41	95	63,8		227	49	41	5	17	67,8	C
Pmsy-129125:5|chr4_1109301_1109317[1109029_1109410]|	195	142	188	66	63	130,8		113	311	210	33	42	141,8	C
Pmsy-55509:3|chr4_1119324_1119339[1118435_1119624]|	424	305	542	512	365	429,6		224	554	604	266	359	401,4	C
msy-554402:5|chr4_1122024_1122045|	0	0	0	0	0	0		0	0	0	0	3	0,6	NC

msy-7177:5|chr4_1122027_1122045|	1815	2099	2300	1545	2879	2127,6		2453	2625	2163	1794	2124	2231,8	NC
Pmsy-20328:3|chr4_1122117_1122132[1121642_1122396]|	1112	737	860	1019	965	938,6		428	1235	1381	1012	705	952,2	C
msy-101516:3|chr4_1122119_1122134|	656	616	536	904	732	688,8		457	1064	1194	1061	669	889	NC
msy-554433:5|chr4_1129149_1129168|	30	71	18	32	35	37,2		33	133	114	13	91	76,8	NC
msy-504393:3|chr4_1146090_1146104|	191	134	118	162	119	144,8		100	188	143	127	93	130,2	NC
Pmsy-35977:5|chr4_1169409_1169431[1166632_1173753]|	2	0	1	1	3	1,4		0	5	2	0	0	1,4	C
msy-451351:5|chr4_1175524_1175544|	1	7	1	5	3	3,4		4	10	6	18	8	9,2	NC
msy-358183:5|chr4_1177579_1177598|	69	74	133	103	106	97		82	101	94	112	97	97,2	NC
msy-101682:3|chr4_1179729_1179745|	308	146	182	329	146	222,2		119	456	322	135	225	251,4	NC
msy-55711:3|chr5_3183_3200|	322	265	1	548	326	292,4		277	3	489	338	2	221,8	NC
Pmsy-196957:5|chr5_9542_9562[9158_10012]|	116	179	141	137	220	158,6		62	153	129	136	129	121,8	C

Pmsy-317111:3|chr5_13565_13580[12900_14081]|	97	46	34	155	44	75,2		47	102	106	58	64	75,4	C
Pmsy-129517:5|chr5_20862_20881[20830_21364]|	291	5	340	5	1	128,4		116	547	5	3	113	156,8	C
msy-101857:3|chr5_30294_30309|	281	287	241	360	522	338,2		395	812	399	641	483	546	NC
msy-729540:3|chr5_30296_30310|	4	3	3	7	4	4,2		6	5	2	8	6	5,4	NC
msy-129615:5|chr5_45748_45772|	56	52	57	77	37	55,8		30	90	74	30	31	51	NC
msy-129740:5|chr5_73117_73138|	6	0	1	0	0	1,4		0	0	4	0	1	1	NC
Pmsy-406423:3|chr5_101139_101154[100199_101937]|	85	46	124	66	88	81,8		47	142	76	62	91	83,6	C
msy-274209:5|chr5_107961_107980|	108	69	88	75	83	84,6		62	172	97	112	82	105	NC
msy-667550:5|chr5_121688_121711|	0	0	0	0	1	0,2		0	0	0	0	0	0	NC
Pmsy-555595:5|chr5_121688_121708[120354_121840]|	12	26	27	27	45	27,4		30	44	20	30	25	29,8	C
msy-452353:5|chr5_155966_155990|	0	0	0	0	0	0		0	1	0	0	0	0,2	NC
Pmsy-20719:3|chr5_164109_164125[163684_164461]|	5759	541	15102	736	770	4581,6		1032	1022	6576	729	7912	3454,2	C

<table>
<tr><td>msy-20720:3|chr5_164111_164126|</td><td>3</td><td>11</td><td>13</td><td>6</td><td>10</td><td>8,6</td><td></td><td>11</td><td>10</td><td>13</td><td>7</td><td>8</td><td>9,8</td><td>NC</td></tr>
<tr><td>Pmsy-406835:3|chr5_164949_164964[164614_165578]|</td><td>75</td><td>41</td><td>71</td><td>54</td><td>100</td><td>68,2</td><td></td><td>68</td><td>126</td><td>127</td><td>48</td><td>152</td><td>104,2</td><td>C</td></tr>
<tr><td>Pmsy-556064:5|chr5_191656_191677[190316_192580]|</td><td>1</td><td>4</td><td>0</td><td>0</td><td>1</td><td>1,2</td><td></td><td>3</td><td>3</td><td>1</td><td>3</td><td>1</td><td>2,2</td><td>C</td></tr>
<tr><td>msy-614283:3|chr5_195328_195343|</td><td>0</td><td>0</td><td>99</td><td>1</td><td>0</td><td>20</td><td></td><td>0</td><td>0</td><td>150</td><td>156</td><td>145</td><td>90,2</td><td>NC</td></tr>
<tr><td>msy-130245:5|chr5_202342_202363|</td><td>4</td><td>0</td><td>5</td><td>3</td><td>2</td><td>2,8</td><td></td><td>3</td><td>11</td><td>3</td><td>3</td><td>2</td><td>4,4</td><td>NC</td></tr>
<tr><td>Pmsy-20851:3|chr5_249490_249504[248317_249627]|</td><td>1073</td><td>852</td><td>889</td><td>1472</td><td>830</td><td>1023,2</td><td></td><td>707</td><td>1760</td><td>1390</td><td>995</td><td>1041</td><td>1178,6</td><td>C</td></tr>
<tr><td>msy-452945:5|chr5_261363_261383|</td><td>64</td><td>25</td><td>191</td><td>137</td><td>164</td><td>116,2</td><td></td><td>15</td><td>34</td><td>160</td><td>33</td><td>63</td><td>61</td><td>NC</td></tr>
<tr><td>Pmsy-359824:5|chr5_310549_310569[310514_310729]|</td><td>68</td><td>76</td><td>41</td><td>128</td><td>82</td><td>79</td><td></td><td>65</td><td>138</td><td>140</td><td>117</td><td>98</td><td>111,6</td><td>C</td></tr>
<tr><td>Pmsy-130669:5|chr5_324540_324560[322899_325509]|</td><td>181</td><td>196</td><td>271</td><td>224</td><td>279</td><td>230,2</td><td></td><td>139</td><td>296</td><td>263</td><td>154</td><td>220</td><td>214,4</td><td>C</td></tr>
<tr><td>msy-615405:3|chr5_342031_342046|</td><td>74</td><td>2</td><td>28</td><td>0</td><td>26</td><td>26</td><td></td><td>41</td><td>1</td><td>54</td><td>43</td><td>42</td><td>36,2</td><td>NC</td></tr>
<tr><td>Pmsy-164873:3|chr5_342033_342048[341794_344196]|</td><td>160</td><td>136</td><td>95</td><td>209</td><td>130</td><td>146</td><td></td><td>162</td><td>231</td><td>171</td><td>119</td><td>230</td><td>182,6</td><td>C</td></tr>
</table>

msy-130727:5|chr5_342426_342444|	6	249	364	1	1	124,2		191	544	379	208	346	333,6	NC
msy-165055:3|chr5_383308_383323|	287	0	1	2	1	58,2		173	332	0	243	1	149,8	NC
msy-21131:3|chr5_424583_424599|	970	657	603	1023	1051	860,8		604	1204	1300	1016	896	1004	NC
msy-77946:5|chr5_446970_446987|	172	320	182	1	2	135,4		295	1	3	195	163	131,4	NC
Pmsy-131099:5|chr5_450964_450984[450332_451768]|	177	127	134	185	187	162		73	220	183	113	194	156,6	C
msy-7999:5|chr5_510288_510307|	597	496	382	579	595	529,8		468	762	731	579	489	605,8	NC
Pmsy-8019:5|chr5_523741_523759[523520_524297]|	1809	9	666	13	577	614,8		3	8	1411	11	8	288,2	C
Pmsy-21275:3|chr5_535182_535198[534907_535917]|	2754	1856	2300	3048	1802	2352		1599	2710	2344	1193	1444	1858	C
Pmsy-360977:5|chr5_548100_548119[546621_548442]|	71	56	66	77	62	66,4		49	112	126	69	56	82,4	C
Pmsy-670354:5|chr5_556163_556184[555601_557455]|	59	35	78	46	36	50,8		29	99	89	43	57	63,4	C
Pmsy-320210:3|chr5_562433_562449[562083_563343]|	9	358	8	610	27	202,4		19	36	19	545	350	193,8	C

Pmsy-508691:3|chr5_566778_566792[564352_567004]|	1	173	2	348	131	131		105	1	2	0	144	50,4	C
msy-103697:3|chr5_667784_667798|	0	1	0	1	3	1		1	2	2	0	2	1,4	NC
Pmsy-166205:3|chr6_3136_3152[2963_3769]|	1	1	334	1	1	67,6		0	533	0	0	356	177,8	C
msy-199695:5|chr6_7978_7995|	188	76	100	143	98	121		69	179	124	73	93	107,6	NC
msy-199775:5|chr6_26567_26588|	77	47	30	70	28	50,4		11	71	111	65	23	56,2	NC
msy-361711:5|chr6_39865_39887|	0	1	5	4	4	2,8		5	1	2	4	7	3,8	NC
msy-166401:3|chr6_48595_48610|	194	126	94	161	70	129		87	170	158	151	71	127,4	NC
msy-21585:3|chr6_67620_67637|	572	387	386	496	303	428,8		185	553	530	365	362	399	NC
Pmsy-618475:3|chr6_80170_80190[80009_81862]|	2	1	0	1	2	1,2		0	4	0	1	2	1,4	C
Pmsy-200093:5|chr6_100718_100739[100467_101950]|	133	112	154	153	139	138,2		65	143	125	79	116	105,6	C
Pmsy-410561:3|chr6_109370_109385[102677_113255]|	105	52	50	80	58	69		48	71	94	42	46	60,2	C
msy-735796:3|chr6_115323_115337|	16	95	254	15	149	105,8		38	22	4	130	0	38,8	NC
msy-200195:5|chr6_125026_125042|	103	112	81	209	97	120,4		109	178	161	179	182	161,8	NC

msy-671967:5|chr6_148685_148713|	0	0	0	1	1	0,4		0	0	0	0	0	0	NC
msy-672409:5|chr6_218771_218791|	1	1	5	1	2	2		0	2	0	1	0	0,6	NC
msy-240791:3|chr6_220016_220031|	28	33	2	21	17	20,2		8	60	43	20	16	29,4	NC
Pmsy-57934:3|chr6_220018_220033[219431_222895]|	434	370	386	417	425	406,4		239	550	630	595	341	471	C
Pmsy-57958:3|chr6_228221_228237[228003_229296]|	375	195	294	457	380	340,2		188	438	446	330	375	355,4	C
Pmsy-511255:3|chr6_234480_234494[233079_234979]|	106	33	97	38	62	67,2		56	105	69	60	53	68,6	C
msy-21933:3|chr6_239388_239402|	1401	1065	567	1509	1312	1170,8		1181	1583	1428	2074	1453	1543,8	NC
msy-21935:3|chr6_239390_239404|	1073	864	584	1467	1123	1022,2		1307	1757	1380	2078	1341	1572,6	NC
Pmsy-37953:5|chr6_244013_244033[243989_244083]|	167	119	189	117	130	144,4		63	172	174	132	115	131,2	C
msy-79224:5|chr6_265823_265844|	142	163	174	117	228	164,8		177	216	147	165	179	176,8	NC
Pmsy-132818:5|chr6_274137_274159[272005_274173]|	0	1	0	1	0	0,4		0	0	0	0	0	0	C

msy-38058:5|chr6_288427_288451|	0	0	0	0	0	0		0	1	0	0	0	0,2 NC
Pmsy-322937:3|chr6_311106_311121[309376_311382]|	80	85	43	130	64	80,4		65	148	106	90	72	96,2 C
msy-79415:5|chr6_333732_333749|	113	329	205	133	428	241,6		464	337	180	113	389	296,6 NC
Pmsy-241859:3|chr6_359648_359662[358916_361761]|	166	302	0	380	270	223,6		64	1	506	332	135	207,6 C
msy-79520:5|chr6_385941_385962|	159	135	103	158	176	146,2		196	260	198	235	265	230,8 NC
Pmsy-412827:3|chr6_389705_389720[389460_389777]|	97	64	37	118	62	75,6		53	104	110	116	91	94,8 C
msy-8749:5|chr6_402897_402918|	2695	1871	1869	2559	2550	2308,8		1638	4052	2778	2157	2018	2528,6 NC
msy-168652:3|chr6_440689_440703|	0	0	0	0	0	0		0	1	0	0	0	0,2 NC
Pmsy-22460:3|chr6_440690_440705[440686_440904]|	506	483	184	1558	522	650,6		700	1433	602	364	817	783,2 C
msy-58807:3|chr6_440692_440706|	313	286	203	850	223	375		404	568	346	165	341	364,8 NC
msy-278714:5|chr6_445570_445593|	1	0	0	0	0	0,2		0	0	0	0	0	0 NC

Pmsy-739132:3|chr6_450317_450331[447975_450749]|	130	0	83	59	22	58,8		15	1	150	42	43	50,2	C
msy-8830:5|chr6_456222_456240|	3136	5281	2349	3147	3733	3529,2		5908	4347	2986	3464	2735	3888	NC
Pmsy-278773:5|chr6_458964_458985[458887_459078]|	1	1	0	1	0	0,6		3	1	0	1	2	1,4	C
Pmsy-133504:5|chr6_476192_476213[475330_478853]|	3	1	2	2	2	2		1	4	0	1	5	2,2	C
msy-58928:3|chr6_487712_487727|	411	206	232	331	199	275,8		98	317	362	217	155	229,8	NC
msy-8889:5|chr6_502611_502629|	815	570	168	833	212	519,6		188	373	371	298	437	333,4	NC
Pmsy-168965:3|chr6_510945_510961[507671_511552]|	343	50	45	237	58	146,6		29	397	466	79	281	250,4	C
Pmsy-22577:3|chr6_513486_513502[513039_514103]|	1618	3208	1058	3767	1017	2133,6		3467	6478	1569	4874	1167	3511	C
msy-38494:5|chr6_518223_518243|	84	5	1	93	74	51,4		4	0	74	1	7	17,2	NC
msy-38495:5|chr6_518618_518638|	11	912	6	1277	1	441,4		562	987	13	838	445	569	NC
msy-279051:5|chr6_524177_524197|	2	0	1	3	3	1,8		2	0	2	1	3	1,6	NC
msy-279144:5|chr6_541957_541975|	54	31	51	13	49	39,6		16	73	70	45	26	46	NC

msy-562197:5|chr6_576831_576852|	0	1	2	2	1	1,2		0	3	1	2	0	1,2	NC
msy-414112:3|chr6_602405_602420|	82	87	116	63	86	86,8		63	122	61	45	49	68	NC
msy-458443:5|chr6_613820_613838|	223	0	2	212	170	121,4		3	297	0	0	0	60	NC
msy-279528:5|chr7_415_434|	5	5	4	2	2	3,6		4	6	2	5	3	4	NC
Pmsy-279633:5|chr7_29096_29115[28970_29369]|	143	73	128	94	92	106		62	192	113	97	119	116,6	C
Pmsy-514738:3|chr7_51636_51651[51437_51794]|	86	63	93	82	79	80,6		86	114	77	80	42	79,8	C
msy-22844:3|chr7_61758_61773|	2577	1349	4	11	6	789,4		3	2150	1920	2	10	817	NC
msy-325341:3|chr7_74655_74670|	66	61	42	87	78	66,8		59	175	108	79	85	101,2	NC
msy-279850:5|chr7_76649_76667|	99	78	103	84	75	87,8		65	147	119	73	76	96	NC
msy-243698:3|chr7_84523_84539|	95	55	67	132	62	82,2		66	139	101	57	68	86,2	NC
msy-80331:5|chr7_85286_85305|	300	254	166	278	337	267		312	501	277	375	214	335,8	NC
msy-515003:3|chr7_87354_87369|	109	59	80	38	89	75		39	112	102	79	81	82,6	NC
Pmsy-364976:5|chr7_90712_90734[88560_90816]|	1	0	0	1	1	0,6		0	0	0	0	0	0	C
Pmsy-134331:5|chr7_93325_93342[92952_94887]|	90	125	54	228	73	114		193	180	124	130	115	148,4	C

Pmsy-9187:5|chr7_96892_96915[95375_97782]|	87	72	69	121	77	85,2		55	131	103	83	89	92,2	C
msy-243867:3|chr7_113158_113174|	102	54	104	128	140	105,6		66	213	147	73	185	136,8	NC
Pmsy-202635:5|chr7_117201_117222[116795_117580]|	2	4	8	3	6	4,6		4	8	10	4	6	6,4	C
msy-365112:5|chr7_118111_118134|	0	0	1	0	0	0,2		0	0	0	0	0	0	NC
Pmsy-9219:5|chr7_118114_118134[117609_118229]|	137	7436	471	3962	10574	4516		6775	9286	148	4268	175	4130,4	C
msy-459249:5|chr7_135721_135739|	25	100	202	102	31	92		33	61	11	80	16	40,2	NC
Pmsy-459252:5|chr7_136279_136295[136099_137048]|	44	93	35	56	70	59,6		150	65	44	63	69	78,2	C
Pmsy-9286:5|chr7_168010_168033[166908_168529]|	14	8	8	13	9	10,4		7	14	15	10	7	10,6	C
msy-563467:5|chr7_170187_170206|	5	7	5	242	5	52,8		9	12	138	13	127	59,8	NC
msy-23025:3|chr7_172653_172669|	565	3	341	681	2	318,4		331	8	5	4	3	70,2	NC
msy-515618:3|chr7_173517_173532|	115	38	91	68	56	73,6		37	99	110	65	43	70,8	NC

msy-326005:3|chr7_191532_191548|	19	21	13	30	19	20,4		11	21	20	15	17	16,8 NC
msy-515729:3|chr7_191534_191550|	288	219	82	313	200	220,4		165	133	268	141	130	167,4 NC
msy-244268:3|chr7_195027_195043|	110	92	285	133	130	150		86	165	135	49	114	109,8 NC
msy-624812:3|chr7_208591_208605|	1	1	0	0	0	0,4		0	2	2	1	1	1,2 NC
Pmsy-170458:3|chr7_214678_214693[212624_214885]|	198	155	265	114	150	176,4		102	333	221	172	267	219 C
Pmsy-59834:3|chr7_219494_219509[218478_219722]|	518	327	287	441	246	363,8		271	561	433	381	327	394,6 C
Pmsy-59916:3|chr7_236617_236633[235884_236925]|	496	108	273	160	76	222,6		35	153	200	231	87	141,2 C
msy-23199:3|chr7_247406_247422|	1612	576	1366	733	1322	1121,8		368	2001	1360	1018	954	1140,2 NC
msy-80819:5|chr7_272680_272699|	0	0	0	0	0	0		2	0	0	0	0	0,4 NC
Pmsy-39279:5|chr7_272680_272697[271897_273498]|	526	251	338	451	164	346		124	456	507	234	177	299,6 C
msy-516500:3|chr7_272833_272848|	100	51	122	140	220	126,6		60	204	211	130	70	135 NC

Pmsy-135111:5|chr7_307287_307306[305520_307811]|	163	134	60	188	94	127,8		125	214	206	215	99	171,8	C
msy-245025:3|chr7_307653_307670|	128	68	142	137	80	111		75	157	148	71	95	109,2	NC
Pmsy-460786:5|chr7_394942_394959[394882_396059]|	0	1	0	231	239	94,2		0	269	215	0	0	96,8	C
msy-9622:5|chr7_398219_398237|	670	453	412	280	415	446		231	526	540	421	217	387	NC
msy-60396:3|chr7_406340_406354|	513	422	1071	560	523	617,8		302	698	561	220	430	442,2	NC
Pmsy-744265:3|chr7_422332_422347[422138_422411]|	79	45	74	81	56	67		36	111	100	46	75	73,6	C
Pmsy-171544:3|chr7_435342_435357[434382_436513]|	79	137	46	249	95	121,2		149	269	94	102	91	141	C
msy-327664:3|chr7_451209_451225|	136	122	93	162	91	120,8		87	168	216	177	111	151,8	NC
Pmsy-23589:3|chr7_466004_466019[465599_466308]|	291	99	122	142	131	157		68	216	175	212	107	155,6	C
msy-366935:5|chr7_470447_470466|	50	63	153	40	104	82		88	141	77	43	70	83,8	NC
Pmsy-517931:3|chr7_470450_470467[470206_471011]|	32	89	136	122	146	105		82	198	145	20	149	118,8	C

msy-39723:5|chr7_490000_490019|	231	184	140	288	398	248,2		242	521	256	448	426	378,6	NC
msy-9794:5|chr8_36799_36815|	668	553	344	1647	532	748,8		1111	1055	824	746	354	818	NC
Pmsy-23714:3|chr8_38408_38423[38168_40632]|	3203	1661	2038	2678	1515	2219		1033	2712	3010	2499	1693	2189,4	C
Pmsy-60753:3|chr8_42375_42391[41644_42807]|	501	338	910	487	645	576,2		252	675	696	539	564	545,2	C
Pmsy-23753:3|chr8_59972_59989[59756_60340]|	2606	2974	1232	4644	2034	2698		3613	4183	4044	3161	3205	3641,2	C
msy-23760:3|chr8_60049_60066|	3076	1477	2600	2881	2266	2460		957	2861	2993	1532	1909	2050,4	NC
msy-108202:3|chr8_60309_60323|	84	292	340	251	113	216		385	477	89	80	111	228,4	NC
Pmsy-565983:5|chr8_67798_67814[67792_67900]|	53	40	56	62	80	58,2		39	118	73	84	91	81	C
Pmsy-23800:3|chr8_75444_75461[74603_75830]|	294	201	211	295	232	246,6		177	435	297	167	206	256,4	C
msy-172242:3|chr8_75446_75463|	485	179	230	384	245	304,6		145	539	470	242	193	317,8	NC
Pmsy-628069:3|chr8_77214_77229[75964_78474]|	59	54	118	47	69	69,4		37	59	76	61	49	56,4	C
Pmsy-566078:5|chr8_84574_84592[82984_88881]|	52	2	41	122	1	43,6		45	121	119	43	98	85,2	C
msy-328634:3|chr8_92051_92067|	73	67	39	38	97	62,8		108	72	99	139	77	99	NC

Pmsy-40062:5|chr8_149455_149474[148998_150559]|	521	218	446	488	371	408,8		155	640	456	171	366	357,6	C
msy-282736:5|chr8_194795_194816|	23	17	11	34	14	19,8		10	17	17	31	25	20	NC
msy-566806:5|chr8_198202_198220|	48	74	49	80	135	77,2		22	123	55	52	104	71,2	NC
msy-24045:3|chr8_199860_199875|	2720	1986	10	5370	1731	2363,4		1735	29	11	4	33	362,4	NC
msy-108792:3|chr8_207570_207585|	159	97	77	1001	658	398,4		651	1103	1041	66	557	683,6	NC
Pmsy-61289:3|chr8_207576_207592[207540_207626]|	347	237	193	249	302	265,6		238	426	299	234	220	283,4	C
msy-519975:3|chr8_208161_208176|	1	50	62	119	0	46,4		34	138	147	1	53	74,6	NC
Pmsy-24101:3|chr8_222373_222389[221964_223379]|	1241	2465	16837	3845	15115	7900,6		27948	2981	1618	485	7735	8153,4	C
msy-108937:3|chr8_248731_248745|	199	207	146	278	357	237,4		306	469	178	450	446	369,8	NC
Pmsy-747468:3|chr8_251159_251175[251040_251703]|	2	4	135	2	110	50,6		1	234	1	98	0	66,8	C
Pmsy-173182:3|chr8_253624_253640[252959_253899]|	180	128	175	197	115	159		87	300	208	135	128	171,6	C

msy-136749:5|chr8_276120_276139|	256	132	228	204	155	195		126	378	242	226	161	226,6	NC
Pmsy-205407:5|chr8_281354_281374[280756_283201]|	107	94	52	89	86	85,6		51	258	144	68	57	115,6	C
Pmsy-24197:3|chr8_283471_283487[283360_284728]|	627	325	389	491	280	422,4		184	554	694	338	271	408,2	C
Pmsy-10096:5|chr8_285363_285383[284758_286397]|	1707	1070	811	789	912	1057,8		625	1810	1239	979	907	1112	C
msy-419984:3|chr8_315956_315972|	0	112	160	155	0	85,4		80	202	0	0	1	56,6	NC
Pmsy-630288:3|chr8_340962_340977[339621_341063]|	85	51	37	86	38	59,4		36	75	96	50	34	58,2	C
Pmsy-680468:5|chr8_348038_348061[346913_348313]|	5	2	2	5	0	2,8		5	3	5	4	3	4	C
msy-24271:3|chr8_350896_350911|	2404	2	1	3700	2204	1662,2		1	4976	11	1586	2	1315,2	NC
msy-10193:5|chr8_363584_363604|	3349	2302	1623	3185	2668	2625,4		2253	4031	4455	4462	2780	3596,2	NC
Pmsy-10206:5|chr8_374765_374783[372088_374838]|	2402	3540	4353	1493	4661	3289,8		5324	2325	1530	926	2821	2585,2	C
msy-173793:3|chr8_403285_403300|	5	19	6	41	61	26,4		34	137	7	1	5	36,8	NC

Pmsy-61741:3|chr8_403288_403302[402837_404663]|	24	24	17	25	37	25,4		15	37	31	19	31	26,6	C
msy-369132:5|chr8_414174_414195|	0	0	1	0	0	0,2		0	0	0	0	0	0	NC
Pmsy-40572:5|chr8_414174_414191[413667_415619]|	502	431	4	5	504	289,2		2	1265	2	388	2	331,8	C
msy-748876:3|chr8_424101_424116|	122	162	1	1	144	86		1	0	195	0	2	39,6	NC
msy-248610:3|chrM_950_965|	28	20	24	43	21	27,2		17	2	0	17	8	8,8	NC
msy-24511:3|chrM_957_974|	111	239	137	1145	599	446,2		658	163	290	654	156	384,2	NC
msy-24515:3|chrM_961_976|	1517	430	353	4363	18	1336,2		2605	7693	1363	968	442	2614,2	NC
msy-24516:3|chrM_962_979|	2227	2805	1318	4772	3068	2838		466	803	3130	3100	327	1565,2	NC
msy-248624:3|chrM_964_980|	66	102	265	148	42	124,6		27	4	206	36	253	105,2	NC
msy-10388:5|chrM_20566_20594|	180	55	88	336	82	148,2		161	406	97	86	91	168,2	NC
msy-82653:5|chrM_20577_20605|	14	41	34	12	31	26,4		29	24	10	21	29	22,6	NC

Supplementary Table S4. Mapping of MalaEx small RNAs to the human genome. Left hand side is the location of the sequence in the *M. sympodialis* genome regions according to the annotation from Zhu Y *et al* (manuscript submitted). Five leftmost columns list feature name, strand, chromosome, start and stop position. Columns 6 to 11 show mapping result to the human genome (strand, chromosome, start position, cigar string in SAM file, and mismatch string in SAM file). Column 12 shows the average count across all 10 samples. Only two features, msy-4613 and msy-10193 (highlighted in grey), have more than 1000 average counts. In these two cases, column 13 shows the corresponding sequence in the human genome. These reads were selected by filtering out reads that map to the human genome more than twice. None of the reads map perfectly as indicated by CIGAR string in the mismatches column that specifies the exact location of the mismatches e.g. MD:Z:15G2T tells us that there are 15 matches, followed by a G substitution, two more matches, then a T substitution.

Malassezia sympodialis					Human							
**NAME**	**STRAND**	**CHR**	**START**	**STOP**	**STRAND**	**CHR**	**START**	**CIGAR**	**SEQ**	**MISMATCHES**	**AVG COUNT**	**Corresponding MSY sequence**
msy-187803	+	3	332185	332207	+	1	63179233	23M	TTGGGGGTTGGAGAGAAGG	MD:Z:15G2T4	**0**	
msy-5674	+	3	1E+06	1318542	+	1	1,18E+08	20M	GTGCGACTCATGGGAGACC	MD:Z:4T1A13		
msy-129740	+	5	73117	73138	-	2	1,28E+08	22M	GGGCCGCCACTACACACGG	MD:Z:0C3A17		
msy-2589	+	2	421196	421215	+	2	1,79E+08	20M	GTTGGGCGGATTAGTCGCA	MD:Z:6A9A3		
msy-187359	+	3	217906	217926	-	3	16247005	21M	GGCGCAGCATAGGATCAAC	MD:Z:3T2A14		
msy-27539	+	1	1E+06	1278293	-	3	21162482	20M	GGCATTACGCCCAATAATGT	MD:Z:3T4C11		
msy-128320	+	4	880442	880462	-	3	54156789	21M	CTTCTCGCTGCCGCGCTTTC	MD:Z:18C0T1	**105.6**	
msy-130727	+	5	342426	342444	+	3	71014607	19M	ATCCGGCGGCGGCGTGTTC	MD:Z:2G1T14		
msy-366935	+	7	470447	470466	+	3	1,43E+08	20M	GATGCGCGTGACGAGCAGC	MD:Z:5A0G13		
msy-268754	+	4	92315	92333	+	4	1610475	19M	CTCGCCGGCGGTAGAGCCG	MD:Z:8A2G7	**136.2**	
msy-23800	-	8	65337	65355	-	4	69241992	18M	CGGGCGCGTGTACAACCT	MD:Z:2A2C12		
msy-279633	+	7	29096	29115	+	4	1,29E+08	20M	ACGTACCCGTGCCTTGTGAT	MD:Z:11G7A0	**111.3**	
msy-131099	+	5	450964	450984	+	5	5652414	21M	CGCCAATGGTACCGCCCTGA	MD:Z:14G0T5	**159.3**	
msy-196957	+	5	9542	9562	-	5	39044034	21M	ACTTCTTAGCCTTCATGGCG	MD:Z:16A0A3	**140.2**	
msy-647127	+	2	831640	831660	-	5	80457120	21M	ATACACAGTCAAGCTCTCCT	MD:Z:5T2G12		
msy-360977	+	5	548100	548119	+	5	1,24E+08	20M	CGCTCAAAGGGTTCCTCAAT	MD:Z:8T9G1	**74.4**	
msy-112123	+	1	519603	519622	+	5	1,33E+08	20M	GCGTACCGTGCCGCAGCTG	MD:Z:3C3A12		
msy-5211	+	3	978180	978201	+	6	1,14E+08	22M	GCCGGGGAGGGGGAGTAG	MD:Z:11A7G2	**126.4**	
msy-332362	+	1	126687	126708	+	6	1,67E+08	22M	AGGTCAGGAAGCCTGAGAG	MD:Z:18T2A0	**0.1**	

msy-277296	+	6	125024	125042	-	7	1,51E+08	19M	CGCCCGTTCCCGACGCACA	MD:Z:5T0C12		
msy-69971	+	2	1E+06	1312031	+	8	73814548	21M	TAGGCGTAGTGGATGGAGA	MD:Z:4T2T13		
msy-637028	+	1	765025	765043	+	8	77688650	19M	TGGTCGCTGGGTCTCGAGA	MD:Z:11A2A4	**56.2**	
**msy-4613**	**+**	**3**	**529378**	**529397**	**+**	**8**	**1,44E+08**	**20M**	**GGACGTGATAGGCTGCGAC**	**MD:Z:15G3T0**	**13537.5**	**GGACGUGAUAGGCUGCGA CC**
msy-345760	+	3	18986	19007	+	9	33453808	22M	GCACAGGGCGAGCAGGGAC	MD:Z:8A11C1	**0**	
msy-29354	+	2	661608	661626	+	9	1,4E+08	19M	GTACGGCGGTGACAGCCTC	MD:Z:1C1G15		
msy-135111	+	7	307287	307306	-	10	1279527	20M	GCCCCACACGACGCGCCCCT	MD:Z:9A0C9	**149.8**	
msy-359824	+	5	310549	310569	-	10	36161769	21M	TCTCACCCCAGCGGTCTCCC	MD:Z:4T7T8		
msy-6950	+	4	960202	960222	-	10	74385436	21M	CAGATTGTCCCAATCGCCTG	MD:Z:4G7G8		
msy-279850	+	7	76649	76667	+	10	97850383	19M	CGGCGCATCGCATCCTCGT	MD:Z:0T7G10		
msy-64600	+	1	803543	803561	-	11	72174121	19M	AGCACGTACCACACGCTTA	MD:Z:4A6A7		
msy-35977	+	4	1E+06	1169431	+	11	1,3E+08	23M	AAGGAGGTGGGACTGGAGT	MD:Z:4T4C13		
msy-80331	+	7	85286	85305	-	12	1,27E+08	20M	TCACCACAACGACGAACACG	MD:Z:9T3T6	**301.4**	
msy-639838	+	1	1E+06	1191485	+	13	1,15E+08	22M	TGAGAGTAAGAATGCAGTG	MD:Z:6G3G11		
msy-172242	-	8	65625	65641	+	14	22249113	18M	GTTGTACACGCGCCCGGA	MD:Z:10T2T4	**311.2**	
msy-26333	+	1	707273	707291	-	14	24458431	19M	GGAAGCCGCTCCGAATACC	MD:Z:0T6C11		
msy-63003	+	1	139147	139167	-	14	55857768	21M	ATCAACCTCGGATCACTGCT	MD:Z:3T4G12		
msy-340380	+	2	287434	287453	-	14	80259817	20M	ACGAGGCACGTGGTTTCAG	MD:Z:6G2C10		
**msy-10193**	**+**	**8**	**363584**	**363604**	**-**	**14**	**93476069**	**21M**	**TGCGTCCAACTCTGCCACCC**	**MD:Z:16T4**	**3110.8**	**CGGGUGGCAGAGUUGGAC GCA**
msy-115519	+	1	1E+06	1488748	-	14	1,05E+08	20M	GCGAGCCACAGCCGGAACA	MD:Z:5G3T10		
msy-435711	+	2	1E+06	1053420	-	15	45448234	20M	ACTCTTCACTTCGCGACGTC	MD:Z:0C11T7	**67.9**	
msy-194933	+	4	699257	699276	+	15	68601437	20M	TAACGGATAAAAGATGCCGT	MD:Z:16G1T1	**98.8**	
msy-535732	+	2	685260	685277	-	16	12833487	18M	GCAGATTGCGACGAGTCG	MD:Z:9T6A1	**66.2**	
msy-667550	+	5	121688	121711	-	16	27360825	24M	CGGAACACCAGGCAGAGGG	MD:Z:3T1G18		
msy-126197	+	4	266775	266794	-	16	69363949	20M	GACGCTCTCGCAGCCCTTGA	MD:Z:17C1G0	**0.2**	
msy-185473	+	2	1E+06	1124046	-	17	55656997	20M	CCTTGTAGGCACCCGTCTTC	MD:Z:5G5G8		
msy-452945	+	5	261363	261383	-	17	71839578	21M	TGTCATCCAGAAGCTCTCCC	MD:Z:2G1T16		
msy-670354	+	5	556163	556184	-	18	65131714	22M	GGCAAGCAGGTGCTCCAGC	MD:Z:18G0T2	**57.1**	
msy-270700	+	4	520983	521001	-	18	73464905	19M	CAGAGCTCGTTCCGCCCAA	MD:Z:18C0	**100.8**	
msy-271357	+	4	668018	668036	-	19	18094119	19M	TGGCTCGACACCTGCTTGC	MD:Z:5G3C9		
msy-341566	+	2	522559	522578	+	19	50276437	20M	CAGACCGAGCGCGTGTTCTG	MD:Z:3C5G10		

msy-72123	+	3	744711	744729	-	21	40032529	19M	CGTGCCTTGGCCGTGGTCG	MD:Z:15C0G2	**142.6**	
msy-7999	+	5	510288	510307	-	22	24459520	20M	GCCTCGTCCGTGCGCGACCT	MD:Z:9C0A9		
msy-358183	+	4	1E+06	1177598	-	22	38141476	20M	ACCTGCGCGAAAAGAGCAT(	MD:Z:6A0G12		
msy-274209	+	5	107961	107980	-	X	32809475	20M	ACCCGAACATCAATGCCAAT	MD:Z:2A1T15		
msy-638155	+	1	926462	926483	-	X	74338053	22M	ATGGCAGCCTGGGTGCCGG(	MD:Z:16A2A2	**62**	
msy-27771	+	1	1E+06	1397285	-	X	78420361	20M	TTGCACCGCACACCCATACC	MD:Z:7T1C10		
